# Xiao‐Chai‐Hu‐Tang Ameliorates Depressive Symptoms via Modulating Neuro‐Endocrine Network in Chronic Unpredictable Mild Stress‐Induced Mice

**DOI:** 10.1111/cns.70290

**Published:** 2025-02-21

**Authors:** Ying Feng, Wenkai Wang, Yingru Zhang, Yuanyuan Feng, Yiyang Zhao, Zhaozhou Zhang, Yan Wang

**Affiliations:** ^1^ Department of Medical Oncology, Shuguang Hospital Shanghai University of Traditional Chinese Medicine Shanghai China; ^2^ School of Integrative Medicine Shanghai University of Traditional Chinese Medicine Shanghai China; ^3^ The Second Clinical Medical College of Guizhou University of Traditional Chinese Medicine Guizhou Province China

**Keywords:** depression, molecular docking, nerve regeneration, network pharmacology, neuroinflammation, RNA sequencing, Xiao‐Chai‐Hu‐Tang

## Abstract

**Objective:**

Xiao‐Chai‐Hu‐Tang (XCHT) has been demonstrated to exert an antidepressant effect during long‐term clinical practices. However, the potential mechanisms of XCHT remain unknown. This study aims to investigate the effect of XCHT on chronic unpredictable mild stress‐induced mice with depressive‐like behaviors and to explore the underlying mechanisms.

**Methods:**

The active compositions and potential related targets of XCHT in the brain were obtained through UPLC‐Q‐TOF‐MS, network pharmacology, and bioinformatics analyses, verified by experimental validation. Then, the protein–protein interaction (PPI), Gene Ontology (GO), Kyoto Encyclopedia of Genes and Genomes (KEGG) analyses, and molecular docking were used to predict the core targets and mechanisms of XCHT on depression. After being treated with XCHT standard decoction, based on enzyme‐linked immunosorbent assay (ELISA), non‐targeted metabolism, targeted LC–MS analyses, RNA‐seq, quantitative RT‐PCR, immunofluorescence, and western blotting were determined to clarify the mechanism of XCHT in the treatment of anxiety and depression disorder.

**Results:**

In total, 166 active ingredients and 525 related targets of XCHT were detected and selected from the network databases. The inflammatory response and metabolism of neurotransmitters were the main related signaling pathways predicted by KEGG enrichment analyses. Behavioral testing shows that XCHT has antidepressant effects, and untargeted metabolomic studies showed it significantly reduced levels of the neurotoxic substance quinoline acid. Combining the results of molecular docking, RNA‐seq, and western blot revealed that XCHT regulated nerve regeneration via BDNF/TrkB/CREB and PI3K/AKT signaling pathways. Immunofluorescence analysis revealed that XCHT downregulated the chronic stress‐induced activation of microglia and astrocytes in the hippocampus.

**Conclusion:**

XCHT exerts antidepressant functions by modulating neuroinflammation and neuroregeneration.

## Graphical abstract

XCHT alleviates depression by anti‐neuroinflammation and rebalancing the HPA axis, neurotransmitter release, and tryptophan metabolic pathway. Meanwhile, XCHT promotes neurogenesis by up‐regulating the BDNF/PI3K/AKT signaling pathway, resulting in harmonizing the neuroendocrine network.

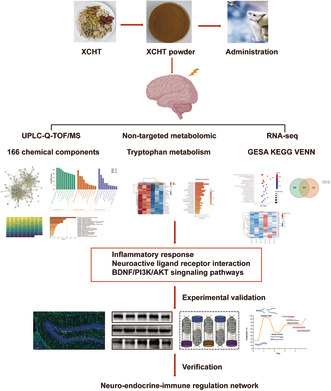

## 1 Introduction

Depression has become a leading cause of disability worldwide, contributing to a range of negative effects on the body, including neurodegeneration, diabetes, cardiovascular disease, and an increased risk of cancer [1, 2, 3]. Acute and chronic stress can result in hypothalamic–pituitary–adrenal (HPA) axis and sympathetic nervous system (SNS) dysregulation, which in turn can contribute to depression [4, 5]. Recently, both clinical and laboratory studies have shown that an imbalance of the HPA axis and SNS, parts of the neuroendocrine and neurotransmitter systems, has associations with brain microenvironment dysregulation and neuroinflammation, leading to alterations in neuronal/synaptic plasticity and neurogenesis [6]. Currently, selective norepinephrine reuptake inhibitors (SNRIs) and selective serotonin reuptake inhibitors (SSRIs) are the first‐line treatments for depression and anxiety [7, 8]. However, many of them have limited efficacy and side effects. As such, it is essential that new, safe, and efficacious treatments are needed for depression.

Traditional Chinese medicine (TCM), has a long history in treating depression and anxiety disorders. Xiao‐Chai‐Hu‐Tang (XCHT), a famous herbal formula initially recorded in “Shang Han Lun”, for the treatment of “Shaoyang syndrome”, has been demonstrated to exert an antidepressant effect in multiple animal models of depression [9]. In several clinical studies, XCHT showed effects on improving cancer patients with depressive disorders and many types of liver diseases without side effects [9, 10, 11]. XCHT is made of seven herbals, including bupleuri radix (*Bupleurum chinense DC*.), ginseng (*Panax ginseng C. A. Mey*.), glycyrrhiza (*Glycyrrhiza uralensis Fisch*.), ginger (*Zingiber officinale Rosc*.), radix scutellariae (*Scutellaria baicalensis Georgi*.), jujubae fructus (*Ziziphus jujuba Mill*.), and pinelliae rhizome ( *Pinellia ternata* *(Thunb.) Breit*.) [12]. The main chemical components reported are saikosaponins, ginsenosides, baicalin, wogonoside, wogonin, and liquiritin. XCHT has a variety of effects, such as anti‐inflammatory, antioxidant, anti‐tumor, and immune system modulation [13, 14]. However, the mechanism of action and specific drug targets remain unclear.

Since TCM has the unique advantages of multi‐components, multi‐targets, and multi‐pathways, combined network pharmacology, molecular docking, and multi‐omics approaches were commonly applied to explore the underlying mechanisms of TCM [15]. Recently, based on network pharmacology and bioinformatics analyses, the potential ingredients and the targets of many different Chinese formulas against depression and other diseases were demonstrated, laying the foundation for further development and clinical application [16, 17, 18]. In a previous study of XCHT, the levels of the monoaminergic system, neurogenesis, and neurotrophin expression in the hippocampus were explored [19]. Likewise, the main chemical components of XCHT were reported to ameliorate neuroinflammation‐induced depressive‐like behavior [20, 21, 22]. However, how XCHT influences the systematic relationship between neuroinflammatory status, neuro‐immune environment, neuroendocrine, neurotransmitter system, and metabolic environment in the brain was unknown. Neuro‐immune environment disorder contributes to up‐regulation of pro‐inflammatory cytokine [23]. It also has been reported that neuroinflammation has the potential to influence tryptophan metabolism, the basis of the depressive central nervous system (CNS) immune milieu [24]. Increased kynurenine (Kyn) metabolized from tryptophan (Try) is a biomarker in the immune dysfunction of depression. Of note, stress‐induced IL‐1, IL‐6, and TNF‐α in the brain have been shown to significantly reduce the expression of brain‐derived neurotrophic factor (BDNF) and PI3K/AKT pathway, which is believed to play a pivotal role in neurogenesis [25, 26]. Meanwhile, the microglia and astrocytes are two main cell types that function as immune surveillance in CNS, and their crosstalk between neurons is known to be a central feature of neurodegenerative diseases. Also, dysregulation of the neuro‐immune environment thereby potentially leads to the dysregulation of the tryptophan metabolic pathway and neuroinflammation [27, 28, 29], affecting the HPA axis and SNS of depression. Therefore, our study aims to uncover anti‐depression targets of XCHT among the numerous drug targets of the brain environment that may have great potential value in future drug development.

In this study, through network pharmacological analysis and molecular docking, we identified the core compounds and targets of XHCT that affected the depressive brain inflammatory environment. Then non‐targeted metabolomic studies, RNA sequencing, and bioinformatics analysis were used to identify drug targets and promising mechanisms. Experimental evidence served to validate predictions. In summary, our results not only provided a novel approach for searching potential targets of XHCT but also demonstrated the mechanistic rationale of XHCT in the treatment of depression.

## 2 Materials and Methods

### 2.1 Preparation and Detection of XCHT

Roots of *Bupleurum chinense DC*. (Chai‐Hu, radix bupleuri, lot number, 20230709), tubers of *P. ternata (Thunb.) Breit*. (Ban‐Xia, pinellia ternate, lot number, 20230617), roots of *P. ginseng C. A. Meyer*. (Ren‐Shen, ginseng, lot number, 2023062410), roots of *Scutellaria baicalensis Georgi* (Huang‐Qin, Scutellaria baicalensis, lot number, 2023080508), tubers of *Zingiber officinale Roscoe* (Sheng‐Jiang, ginger, lot number, 2023042808), roots of *G. uralensis Fisch*. (Gan‐Cao, glycyrrhiza, lot number, 2023061907) and fruits of *Ziziphus jujuba Mill*. (Da‐Zao, jujubae fructus, lot number, 20230418) were purchased from Chinese Pharmacy, Shuguang Hospital Affiliated to Shanghai University of Traditional Chinese Medicine (Shanghai, China). XCHT was prepared using a water decoction as described previousl [9]. All herbs (Chai‐Hu:48 g, Ban‐Xia:18 g, Huang‐Qin:18 g, Ren‐Shen:18 g, Shen‐Jiang:18 g, Gan‐Cao:18 g, Da‐Zao: 20 g) were weighed and soaked in 900 mL water for 1 h. Then, the herbs and water were brought to a boil and simmered for 30 min. Following this, the upper layer of the liquid was removed (500 mL) and pure water was added to the residue. The mixture was stirred for 45 min, then filtered and concentrated by a rotary evaporator. The remaining solution was freeze‐dried and stored at 4°C. The final lyophilized powder yield was 36.22 g. Weighed 5 g lyophilized powder and dissolved it in 20% methanol–water, ultrasonic extracted for 30 min, then vortexed for 5 min. Finally, the sample was centrifuged at 12,000 rpm for 5 min, and a 2 μL aliquot was filtered with a 0.22 μm membrane and injected into the UPLC‐Q‐TOF‐MS system for data acquisition.

Analytes were separated by the Waters H‐Class UPLC system (Waters, USA) using a Waters CORTECS@ UPLCC18 (2.1 × 100 mm, 1.6 μm) at 30°C. The gradient solvent system consisted of acetonitrile (A) and 0.1% formic acid‐water (B) as follows: 0–5 min, 5%–10% A; 5–30 min, 10%–30% A; 30–45 min, 30%–50% A; 45–48 min, 50%–75% A; 48–51 min, 75%–95% A, which were delivered at a flow rate of 0.3 mL/min, UV detection at 190–400 nm, and an injection volume of 2 μL. MS data were acquired using the AB Sciex Triple TOF 4600 system (SCIEX, USA) equipped with an electrospray ionization (ESI) source. Analyte detection was carried out using MRM in a positive/negative mode. The mass spectrometry parameters are shown in Table S1. The data acquisition software is Analyst TF 1.7.1, and the data processing software is Peakview 1.2. The mass spectrometry data is initially matched with the Natural Products HRMS/MS Spectral Library 1.0 database, and the compounds are prioritized based on the score information of each chromatographic peak. Subsequently, the compounds are further verified through the analysis of primary and secondary information associated with each chromatographic peak.

### 2.2 Bioactive Compounds and Their Related Targets of XCHT

To fully delineate the bioactive components of XCHT absorbed into the serum and acting on brain tissue, the UPLC‐Q‐TOF‐MS was adopted to detect the bioactive compounds of XCHT in the mouse hippocampus. To fill in missing values of absorbed compounds resulting from insufficient mass spectrometry techniques, we utilized the Traditional Chinese Medicine System Pharmacology (TCMSP, https://old.tcmsp‐e.com/tcmsp.php) database. Using the integrative absorption, distribution, metabolism, and excretion model, we assessed oral bioavailability (OB) as the percentage of an orally administered drug that reaches the systemic circulation, while drug‐likeness (DL) was defined as the similarity of a compound to a known drug, and blood–brain barrier (BBB) characterized by the presence of tight intercellular junctions between continuous non‐fenestrated endothelial cells [30], which normally function to limit the passage of protein and potential diagnostics into the brain parenchyma [31]. OB ≥ 30%, DL ≥ 0.18, and BBB > −0.3 are adopted as the candidate active compounds screening threshold [32]. Besides, the UPLC‐Q‐TOF‐MS was adopted to detect the bioactive compounds of XCHT in the mouse hippocampus, which also added direct evidence about the substances of XCHT absorbed and passed through the BBB. For bioactive components that have not been retrieved, based on the Canonical SMILES of components, we use Swiss Target Prediction (http://www.swisstargetprediction.ch/) and similarity ensemble approach (SEA, https://sea.bkslab.org/) to query the target protein of the component.

### 2.3 Search for Putative Targets of Depression

Using the publicly available genetic database of human clinical disease, such as DisGeNET (https://www.disgenet.org/), Online Mendelian Inheritance in Man (OMIM, https://omim.org/), GeneCards (https://www.genecards.org/), Drugbank (https://go.drugbank.com/), and the Therapeutic target database (TTD, http://db.idrblab.net/ttd/), to collect clinically variant genes or therapeutic targets associated with depression. In this study, “depression” or “anxiety” was used as the search keywords for stress‐related diseases, and all search dates were 2022‐07‐15, and they were obtained after excluding coincidences. The gene names and UniProt IDs corresponding to the targets associated with XCHT and depression were obtained by importing them into the UniProt database (https://www.uniprot.org/) and limiting the search to ‘ *Homo sapiens* ’.

### 2.4 Construction of the Protein–Protein Interaction (PPI) Network

To identify genes that are both XCHT and depression‐related, the Venny database (version 2.1, https://bioinfogp.cnb.csic.es/tools/venny/) was used, and the genes in the intersection are the Potential targets for XCHT in the treatment of depression. Next, we constructed a protein–protein interaction (PPI) network using STRING (version 11.5, http://string‐db.org/) with a focus on “ *Homo sapiens* ” and a confidence score > 0.7. We imported the results into Cytoscape (version 3.9.1) software and analyzed the network using the Network Analyzer plugin. We calculated the topological properties of each node, including “degree,” “betweenness centrality,” “closeness centrality,” and “average shortest path length,” to identify putative nodes of topological importance. Generally, nodes with higher degrees, betweenness centrality, or closeness centrality are more important in the network.

### 2.5 Gene Ontology (GO) and Kyoto Encyclopedia of Genes and Genomes (KEGG) Enrichment Analysis

GO and KEGG enrichment analyses were performed using the Metascape database (https://metascape.org/gp/index.html#/main/step1). The GO enrichment analysis included biological process (BP), cellular component (CC), and molecular function (MF) terms of gene function.

### 2.6 Molecular Docking

To further verify the drug‐target relationship, we used the Surflex‐DOCK docking tool to conduct key components with high degree scores obtained in the “ingredient‐drug‐target” relationship network with key targets in the PPI network. First, the RCSB PDB Protein Database (https://www. rcsb.org/) was employed to obtain a high‐resolution crystal structure (Å < 2.50) of the key target, and PubChem compounds (https://www.ncbi.nlm.nih.gov/pccompound) were employed to obtain a three‐dimensional (3D) structure of core components. Then, in order to optimize the molecular structure and improve the accuracy of molecular docking, we used Open Babel 2.4.1 (http://openbabel.org) [33] to remove the original ligand, remove the water molecules, separate the proteins, add hydrogen atoms, and convert from its native format to the pdbqt format. Molecular docking studies are performed using Autodock Vina 1.2.0 [34]. The docking process is calculated using a genetic algorithm. All docked run options are defaults. Finally, the docking results with the highest scores were visualized using PyMoL 2.5.4 [35].

### 2.7 Animals

Male C57BL/6J mice (*n* = 100, 6–8 weeks) were purchased from the Shanghai SLAC Laboratory Animal Co. Ltd. (Permission No: SCXK 2018–0006). All mice were individually housed in a specific pathogen‐free condition (22°C ± 0.5°C, 50% ± 5% humidity, and a 12 h light/12 h dark cycle) and maintained with free access to sterile water and chow diet. The animal protocols were carried out strictly following the National Guidelines for Animal Usage in Research (China) and were approved by the Ethics Committee of Shanghai University of Traditional Chinese Medicine (PZSHUTCM2100409007). The mice were randomly grouped in individual experiments (*n* = 20 per group). All mice were allowed 1 week of acclimation to the housing facilities before being randomly assigned to experimental groups. According to our previous study [9], the model (orally treated with saline), fluoxetine (selective 5‐HT reuptake inhibitor, Sigma, USA, 2.6 mg/kg/day), XCHT‐L (10.27 g/kg/day), and XCHT‐H (20.54 g/kg/day) were subjected to a 4‐week period of CUMS. The saline was given to mice with gavage which did not suffer CUMS.

### 2.8 CUMS Procedure and Behavior Test

Here, we used the CUMS approach to be consistent with previous publications [36, 37]. In addition to the control group, the mice in the other four groups were randomly exposed to different stressors: cold swimming for 3 min (at 0°C), water or food deprivation for 24 h, level shaking for 15 min, cage tilting for 24 h, tail nip for 1 min (1 cm from the end of the tail), wet bedding for 24 h, and inversion of the light/dark cycle for 24 h. After 4 weeks, depressive phenotypes were assessed by the sucrose preference test (SPT), open‐field test (OFT), and the tail suspension test (TST).

#### 2.8.1 Sucrose Preference Test

After 4 weeks of chronic unpredictable mild stress, animals were monitored using the sucrose preference test (SPT) to testify if anhedonia existed, a core symptom of depression [38]. Before test day, animals were habituated to 1% sucrose (Sigma‐Aldrich) for three consecutive days with two water bottles. The placement of the two water bottles was reversed each day. Mice were deprived of water for 15 h before the test day. On the test day, all animals were separated into five groups provided with a two‐bottle choice for 8 h, and the preference for sucrose was calculated (sucrose preference = 100 × (sucrose−water)/total).

#### 2.8.2 Open‐Field Test

The open‐field test was conducted with minor modifications based on a previous report [39]. Briefly, mice were placed in a center open‐field apparatus measuring 50 cm × 50 cm × 40 cm, which was divided into four squares. The behavior of the mice was recorded for the next 5 min using a camera, and the total distance covered by the mice was documented.

#### 2.8.3 Tail Suspension Test

The TST is a classical behavioral test widely used to evaluate the degree of depression and response to antidepressant treatments [40]. In the TST, all the mice were hung upside down, 50 cm above the test box, with adhesive tape affixed 1 cm from the tip of the tail. A six‐minute behavior test was conducted with an adaptation period of the first 2 min and a four‐minute behavior was recorded. To evaluate the degree of depression, the immobility time of mice in the four‐minute behavior test was measured.

### 2.9 Tissue Collection

After the behavioral tests, all animals were anesthetized by intraperitoneal injection of a sodium pentobarbital solution. The hippocampal tissues were quickly dissected on ice‐cold plates and rinsed three times with cold PBS, then transferred to liquid nitrogen and stored at −80°C for subsequent biochemical analysis.

### 2.10 Non‐targeted Metabolomic Studies and Measurement of Neurotransmitters

Buffers consisted of 100% acetonitrile for mobile A, and 0.1% NH_4_OH/20 mM CH_3_COONH_4_ in water for mobile B. The gradient ran from 85% to 30% A in 20 min, followed by a wash with 30% A and re‐equilibration at 85% A. Metabolites were identified on the basis of exact mass within 5 ppm and standard retention times. Tracing experiments were performed at the Shanghai Jiao Tong University School of Medicine. After washing twice with cold PBS, metabolites were extracted from the hippocampus using the methanol extraction method. Briefly, 1 mL of pre‐cooled 80% methanol was added to each sample, which was then kept at −80°C for 20 min. Subsequently, samples were centrifuged at 4°C for 5 min at 14,000 rpm, and the supernatants were collected and normalized based on the tissue amount. Targeted LC–MS analyses were conducted on a Q Exactive Orbitrap mass spectrometer (Thermo Fisher Scientific, USA) coupled with a Vanquish UPLC system (Thermo Fisher Scientific, USA) operating in polarity‐switching mode. Metabolites were separated using a Sequant ZIC‐HILIC column (2.1 mm i.d. × 150 mm, Merck) at a flow rate of 150 μL/min. Analyte detection was performed using the multiple reaction monitoring (MRM) technique in positive mode.

The Metabolite Set Enrichment Analysis (MSEA) was then performed to analyze various metabolite expression pathways. Finally, differential metabolites were classified using enrichment analysis and a heatmap on the MetaboAnalyst 5.0 platform. The expression levels in the hippocampus of three neurotransmitters and five relative metabolite quantitations were performed based on the peak area for each metabolite.

### 2.11 RNA Sequencing (RNA‐Seq) and Bioinformatics Analysis

After mice were sacrificed by decapitation the fresh hippocampal tissues were separated out, frozen in liquid nitrogen, and stored at −80°C for RNA‐seq. High‐throughput sequencing of transcriptional profiles was performed by Shenzhen Huada Gene Technology Co. Ltd. And samples with total RNA amount > 10 μg, concentration > 100 ng/μL, RIN/RQN ≥ 7, 28S/18S ratio ≥ 1.0 were selected for transcriptome construction. The raw sequence data obtained from sequencing were processed with quality control to obtain clean data. Unless otherwise stated, all RNA‐Seq data is presented in Log10 (1 + TPM), which we refer to as Log (TPM) henceforth. DEGs were screened by comparing the model group with the control group and comparing the XCHT group with the model group, with the following criteria: FPKM > 0.1, fold change ≥ 2 or FDR ≤ 0.001, *p* < 0.05 or Adjusted *p* value ≤ 0.001 (3–4 mice per group). Differential analysis of RNA‐Seq data was performed using DESeq2 version 1.22.2.

### 2.12 Enzyme‐Linked Immunosorbent Assay

After centrifugation, the supernatant was taken for the determination of HPA axis hormones and norepinephrine levels by enzyme‐linked immunosorbent assay (ELISA). Two HPA axis hormones: cortisol (CORT), adrenocorticotropic hormone (ACTH), and norepinephrine levels were measured using ELISA (Jianglaibio JL11918, CUSABIO CSB‐E06874m, and CSB‐E07870m, respectively).

### 2.13 Quantitative RT‐PCR

Quantitative reverse‐transcription PCR (qRT‐PCR) was performed to quantify inflammatory cytokines in the hippocampus and sequencing as described following: IL1β forward PCR‐primer: CACTACAGGCTCCGAGATGAACAAC, IL1β reverse PCR‐primer: TGTCGTTGCTTGGTTCTCCTTGTAC, IL‐6 forward PCR‐primer: CTCCCAACAGACCTGTCTATAC, IL‐6 reverse PCR‐primer: CCATTGCACAACTCTTTTCTCA, TNF‐α forward PCR‐primer: ATGTCTCAGCCTCTTCTCATTC, TNF‐α reverse PCR‐primer: GCTTGTCACTCGAATTTTGAGA. Specific qRT‐PCR procedures with a QuantStudio 7 Flex Real‐Time PCR System (Life Technologies, Gaithersburg, Maryland) applied were reported in our previous study. All operations were performed according to the manufacturer's instructions. The comparative cycle threshold (CT) was applied to calculate the relative expression of mRNAs. The 2^−ΔΔ*C*q^ method was applied to calculate and quantify the relative expression of mRNAs, and GAPDH was used as an endogenous control.

### 2.14 Immunofluorescence

Before sacrifice, all the mice were intraperitoneally injected with BrdU (Beyotime, ST1056) 50 mg/kg every 2 days for 10 consecutive days. For perfusion fixation, all mice were anesthetized and perfused through the aorta with 4% paraformaldehyde. After being fixed with paraformaldehyde for 24 h. Then, the paraffin‐embedded samples were cut to a thickness of 4 μm and subjected to antigen retrieval. Finally, brain specimens were rinsed three times with PBS and coverslipped with Fluoroshield Mounting Medium containing DAPI (Abcam, ab104139). The primary antibody for glial fibrillary acidic protein (anti‐GFAP, CST, 1:600, 3670S), IBA‐1 (anti‐IBA‐1, CST, 1:800, 17198S), NeuN (anti‐NeuN, Servicebio, 1:1500, GB11138‐100), and BrdU (anti‐BrdU, Proteintech, 1:300, 66,241‐1‐Ig) was prepared in 1xPBS containing 0.01% triton‐x (Sigma) and 5% BSA. All brain slices were pictured by Leica VERSA8 fluorescence scanners.

### 2.15 Western Blotting

In this study, hippocampal tissues weighing 25 mg were manually cut into 1 mm2 pieces using eye scissors. The tissue was then homogenized with an ultrasonic crusher (SONICS & MATERIALS INC., USA) in 300 μL of cold RIPA lysis buffer (Epizyme, PC101) supplemented with phosphatase inhibitor (TargetMol, C0002) and protease inhibitor (TargetMol, C0001) for 2 min. After the total protein content was measured using a BCA kit (Beyotime, P0010S), 5× SDS‐PAGE Sample Loading Buffer (Beyotime, P0015L) was added to the samples, and the proteins were denatured by boiling in water for 10 min, and 30 μg total protein was loaded per sample. Subsequently, 10% SDS‐PAGE was used to separate the proteins by molecular weight, and PVDF membranes (0.45 μm, Millipore) were used to transfer them. The membranes were then blocked in 5% skim milk diluted in Tris‐buffered saline with 1% Tween‐20 (TBST) for 1 h and incubated with primary antibodies including rabbit monoclonal anti‐p‐AKT (1:1000, CST, 4060S), rabbit monoclonal anti‐AKT (1:1000, CST, 9272S), rabbit monoclonal anti‐PI3K (1:1000, CST, 4249S), rabbit monoclonal anti‐CREB (1:1000, CST, 9197S), rabbit monoclonal anti‐p‐CREB (1:1000, CST, 9198S), rabbit monoclonal BDNF (1:1000, Proteintech, 28,205‐1‐AP), rabbit monoclonal TrkB (1:1000, CST, 4603S) and mouse monoclonal anti‐GAPDH (1:4000, CST, 2118S) at 4°C overnight. The membranes were washed three times with TBST (10 min per wash) and then incubated with horseradish peroxidase‐labeled goat anti‐rabbit IgG (1:2000, Beyotime, A0208) for 1 h at room temperature. Finally, the membranes were visualized using an enhanced chemiluminescence kit (Millipore, WBKLS0500) and a chemiluminescence imaging system (ChemiScope 6100, Clinx Science Instruments Co. Ltd., China).

### 2.16 Statistical Analysis

The data are presented as the mean ± standard error of the mean (SEM). The statistical analysis was carried out by one‐way analysis of variance (ANOVA) with the Bonferroni post hoc test and independent samples t‐test after being assessed with the Shapiro–Wilk test for normal distribution. The non‐parametric Mann–Whitney test was applied for data that were not normally distributed. All statistics were analyzed by GraphPad Prism 8.0.1 (GraphPad Software, San Diego, CA, USA). Statistical significance was considered when *p* < 0.05.

## 3 Results

### 3.1 Compounds Analysis of XCHT

**FIGURE 1:**
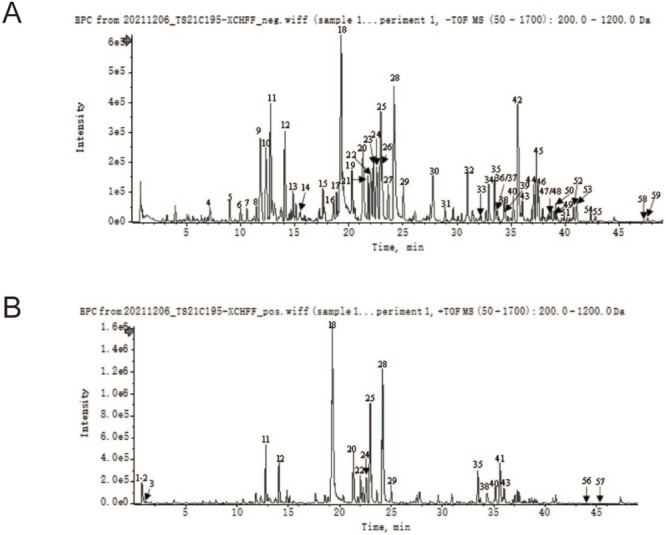
Signature chromatography, mass spectrometry of XCHT compounds. (A) UPLC‐HRMS base peak chromatogram (BPC)‐negative ion mode; (B) UPLC‐HRMS BPC‐positive ion mode.

As shown in Figure 1 and Table 1, a total of 59 characteristic compounds of XCHT were successfully identified, which include flavonoids, saponins, and phenolic acids mainly derived from ginseng, scutellaria baicalensis, radix bupleuri, and glycyrrhiza. Besides, In mouse brain tissue, 38 herbal‐derived monomers were identified as potential pharmacodynamic components for absorption into the brain by UPLC‐Qtof‐MS (Figures S1 and S2; Table S2).

**TABLE 1:** Mass spectrometry parameters of the 59 components in XCHT and their sources.

No.	Tim (min)	Adduct ion	Molecular formula	Molecular weight	Name	MS/MS data	Source
1	0.97	[M + H]^+^	C_10_H_12_N_5_O_6_P	329.05	cAMP	330.0597; 136.0617	*Fructus Ziziphi Jujubae*
2	0.98	[M + H]^+^	C_9_H_8_O_3_	164.04	4‐Hydroxycinnamic acid	123.0444; 119.0503; 95.0489; 91.0534	*Pinellia ternata*
3	1.03	[M + H]^+^	C_10_H_13_N_5_O_5_	283.09	Guanosine	152.0564; 135.0297	*Fructus Ziziphi Jujubae*
4	7.17	[M‐H]^−^	C_15_H_12_O_7_	304.06	Ganhuangemin	303.0474; 217.0487; 177.0180; 149.0231; 125.0239	*Scutellaria baicalensis*
5	9.00	[M + FA‐H]−	C21H36O11	464.23	Not identified	509.2319; 463.2179; 331.1758; 191.0577; 161.044	Unable to identify
6	10	[M‐H]^−^	C_15_H_10_O_7_	302.04	Viscidulin I	301.0344; 283.0232; 257.0465; 175.0035; 149; 0248; 125.0251	*S. baicalensis*
7	10.58	[M + H]^+^	C_26_H_28_O_14_	564.14	Schaftoside	565.1567; 574.1455; 529.1324; 511.1233	*Glycyrrhiza uralensis*
8	11.49	[M‐H]^−^	C_21_H_22_O_9_	418.13	Neoliquiritin	255.0662; 135.0085; 119.0500	*G. uralensis*
9	11.83	[M‐H]^−^	C_21_H_22_O_9_	418.13	Liquiritin	417.1154; 255.0644; 135.0083; 119.0497	*G. uralensis*
10	12.35	[M‐H]^−^	C_26_H_30_O_13_	550.17	Liquiritin apioside	549.1648; 255.0670; 135.0089	*G. uralensis*
11	12.76	[M‐H]^−^	C_26_H_28_O_13_	548.15	Chrysin 6‐C‐glucoside‐8‐C‐arabinoside	547.1459; 487.1237; 457.1136; 427.1021; 367.0807; 337.0701	*S. baicalensis*
12	14.08	[M‐H]^−^	C_26_H_28_O_13_	548.15	Chrysin 6‐C‐D‐arabinoside‐8‐C‐glucoside	547.1479; 457.1149; 427.1040; 367.0825; 337.0719	*S. baicalensis*
13	14.85	[M‐H]^−^	C_26_H_28_O_13_	548.15	Chrysin 6‐C‐hexoside‐8‐C‐pentoside	547.1475; 457.1136; 427.1027; 367.0808; 337.0701	*S. baicalensis*
14	15.51	[M‐H]^−^	C_21_H_20_O_9_	416.11	Puerarin	415.1037; 295.0610; 267.0654	*Fructus Ziziphi Jujubae*
15	17.62	[M‐H]^−^	C_22_H_20_O_12_	476.1	5,7,2′‐trihydroxy‐6‐methoxyflavone‐7‐O‐glucuronide	475.0903; 299.0565; 284.0326	*S. baicalensis*
16	18.57	[M + FA‐H]^−^	C_22_H_22_O_9_	430.13	Ononin	299.0541; 267.0644; 252.0422	*G. uralensis*
17	18.86	[M‐H]^−^	C_21_H_22_O_9_	418.13	Isoliquiritin	417.1200; 255.0669; 148.0168; 135.0097; 119.0506	*G. uralensis*
18	19.23	[M‐H]^−^	C_21_H_18_O_11_	446.08	Baicalin	445.0779; 269.0453; 251.0339; 223.0394	*S. baicalensis*
19	20.3	[M‐H]^−^	C_21_H_20_O_11_	448.1	Dihydrobaicalin	447.0938; 271.0620; 243.0670; 113.0225	*S. baicalensis*
20	21.32	[M‐H]^−^	C_21_H_18_O_11_	446.08	Wogonin5‐O‐β‐D‐glucoside	269.0456; 197.0606	*S. baicalensis*
21	21.78	[M + FA‐H]^−^	C_42_H_72_O_14_	800.49	Ginsenoside Rg1	845.4980; 799.4845; 637.4321; 475.3797	*Panax ginseng*
22	22.04	[M‐H]^−^	C_22_H_20_O_12_	476.1	5,7,8‐trihydroxy‐6‐methoxyflavone‐7‐O‐glucuronide	475.0977; 299.0569; 284.0339	*S. baicalensis*
23	22.27	[M‐H]^−^	C_21_H_18_O_11_	446.08	Norwogonin 8‐O‐β‐D‐glucuronide	445.0768; 269.0443; 197.0595	*S. baicalensis*
24	22.58	[M‐H]^−^	C_21_H_18_O_10_	430.09	Chrysin‐7‐O‐β‐D‐glucoronide	429.0824; 253.0493; 209.0597; 175.0233	*S. baicalensis*
25	22.93	[M‐H]^−^	C_22_H_20_O_11_	460.1	Oroxylin A 7‐O‐glucuronide	459.0966; 283.0614; 268.0385	*S. baicalensis*
26	23.1	[M‐H]^−^	C_22_H_20_O_12_	476.1	Diosmetin 7‐O‐β‐D‐glucuronide	475.0879; 299.0569; 284.0322	*S. baicalensis*
27	23.64	[M‐H]^−^	C_21_H_18_O_11_	446.08	Baicalein 6‐O‐β‐D‐glucuronide	445.0774; 269.0449; 241.0496	*S. baicalensis*
28	24.17	[M‐H]^−^	C_22_H_20_O_11_	460.1	Wogonoside	459.0949; 283.0611; 268.0378	*S. baicalensis*
29	25.04	[M‐H]^−^	C_23_H_22_O_12_	490.11	5,7‐Dihydroxy‐8,2′‐dimethoxyflavone7‐O‐β‐D‐glucuronide	313.0714; 298.0468; 283.0229; 211.0380	*S. baicalensis*
30	27.78	[M‐H]^−^	C_15_H_10_O_5_	270.05	Baicalein	269.0455; 241.0498; 223.0390; 195.0448	*S. baicalensis*
31	28.89	[M + FA‐H]^−^	C_42_H_72_O_14_	800.49	Ginsenoside Rf	799.4876; 637.4347; 475.3799	*P. ginseng*
32	31	[M‐H]^−^	C_42_H_62_O_17_	838.4	Licorice saponin G2	837.3946; 792.4241; 351.0542	*G. uralensis*
33	32.22	[M + FA‐H]^−^	C_36_H_62_O_9_	638.44	Ginsenoside F1	683.4373; 637.4309; 475.3805	*P. ginseng*
34	32.94	[M + FA‐H]^−^	C_54_H_92_O_23_	1108.6	Ginsenoside Rb1	1107.6034; 945.5485	*P. ginseng*
35	33.48	[M‐H]^−^	C_42_H_62_O_17_	838.4	Yunganoside K2	837.3971; 351.0581	*G. uralensis*
36	33.7	[M + FA‐H]−	C_53_H_90_O_22_	1078.59	Ginsenoside Rb2	1123.5979; 1077.5898; 945.5509	*P. ginseng*
37	33.73	[M‐H]^−^	C_48_H_76_O_19_	956.5	Ginsenoside Ro	955.4977; 793.4429; 731.4371	*P. ginseng*
38	34.38	[M‐H]^−^	C_16_H_12_O_5_	284.07	Wogonin	283.0627; 268.0374; 239.0338; 184.0517; 163.0028	*S. baicalensis*
39	34.48	[M + FA‐H]^−^	C_53_H_90_O_22_	1078.59	Ginsenoside Rb3	1123.6026; 1077.5988; 945.5531	*P. ginseng*
40	35.2	[M‐H]^−^	C_42_H_62_O_17_	838.4	Uralsaponin U	837.3927; 351.0581	*G. uralensis*
41	35.57	[M + H‐H_2_O]^+^	C_17_H_26_O_4_	294.18	6‐Gingerol	177.0910;162.0673;145.0635;137.0592	*Ginger*
42	35.64	[M‐H]^−^	C_42_H_62_O_16_	822.4	Glycyrrhizin	821.4006;351.0571;193.0350	*G. uralensis*
43	36.05	[M‐H]^−^	C_19_H_18_O_8_	374.1	Skullcapflavone II	373.0914;358.0686;343.0457;328.0220;300.0271	*S. baicalensis*
44	37.09	[M + FA‐H]^−^	C_42_H_68_O_13_	780.47	Saikosaponin A	825.4637;779.4657;617.4091	*Radix bupleuri*
45	37.34	[M‐H]^−^	C_42_H_62_O_16_	822.4	Uralsaponin B	821.4049;351.0571;193.0354	*G. uralensis*
46	37.53	[M + FA‐H]^−^	C_42_H_68_O_13_	780.47	Saikosaponin B2	825.4680; 779.4622; 617.4091	*R. bupleuri*
47	38.72	[M + FA‐H]^−^	C_42_H_68_O_13_	780.47	Saikosaponin B1	825.4713; 779.4644; 617.4086	*R. bupleuri*
48	38.73	[M + FA‐H]^−^	C_44_H_70_O_14_	822.48	2″‐O‐acetylsaikosaponin A	867.4850; 821.4695; 779.4597; 761.4490; 617.4086	*R. bupleuri*
49	38.98	[M + FA‐H]^−^	C_44_H_70_O_14_	822.48	4″‐O‐acetylsaikosaponin A	867.4874; 821.4711; 779.4588; 761.4485; 617.4053	*R. bupleuri*
50	39.13	[M + FA‐H]^−^	C_42_H_68_O_13_	780.47	Saikosaponin D	825.4631; 779.4597; 617.4057	*R. bupleuri*
51	39.91	[M + FA‐H]^−^	C_42_H_68_O_12_	764.47	Saikosaponin M	809.4778; 763.4716; 601.4154	*R. bupleuri*
52	40.77	[M + FA‐H]^−^	C_44_H_70_O_14_	822.48	3″‐O‐acetylsaikosaponin D	867.4760; 821.4692; 779.4589; 761.4481; 617.4083	*R. bupleuri*
53	41.07	[M + FA‐H]^−^	C_44_H_70_O_14_	822.48	4″‐O‐acetylsaikosaponin D	867.4841; 821.4672; 779.4784; 761.4559; 617.4118	*R. bupleuri*
54	42.36	[M + FA‐H]^−^	C_42_H_72_O_13_	784.5	Ginsenoside F2	829.5007; 783.4946; 621.4371; 459.3849	*P. ginseng*
55	42.72	[M + FA‐H]^−^	C_44_H_70_O_14_	822.48	6″‐O‐acetylsaikosaponin D	867.4767; 821.4711; 779.4605; 761.4481; 617.4070	*R. bupleuri*
56	44.08	[M + H]^+^	C_17_H_24_O_3_	276.17	6‐Shogaol	137.0594; 122.0362	*Ginger*
57	45.15	[M + H]^+^	C_17_H_24_O_3_	276.17	8‐Shogaol	137.0606; 122.0359	*Ginger*
58	47.29	[M + FA‐H]^−^	C_42_H_70_O_12_	766.49	Ginsenoside Rg5	811.4870; 765.4800; 603.4266; 161.0454	*P. ginseng*
59	47.62	[M + FA‐H]^−^	C_42_H_70_O_12_	766.49	Ginsenoside Rk1	811.4854; 765.4841; 603.4311; 161.0458	*P. ginseng*

### 3.2 Bioactive Compounds and Related Targets Identified in XCHT

In total, 166 chemical components, prototype compounds, and their metabolites (38 components were identified by UPLC‐Q‐TOF‐MS and 128 components were from TCMSP) were screened and identified as the main bioactive substances of XCHT through the BBB after removing duplicates (Table 2). Then, 525 XCHT bioactive‐compound‐related target genes were collected.

**TABLE 2:** The main bioactive substances of XCHT through the BBB.

Tibetan medicine	Amount of active ingredients
*Radix bupleuri*	11
*Pinellia ternata*	6
*Ginseng*	15
*Glycyrrhiza*	86
*Chinese date*	9
*Ginger*	2
*Scutellaria baicalensis*	29
De‐duplication	8

As shown in Figure S3, 697 nodes and 3938 edges were contained in the “Herbs‐Compounds‐Targets” network of XCHT, which indicated that as the main components of XCHT, herbal medicines such as ginseng, Scutellaria baicalensis, Bupleurum chinense, and licorice contain a large number of chemical components and their corresponding targets, which may be the main components that contribute to the efficacy of the compound.

### 3.3 Construction of a PPI Network of XCHT‐Depression

**FIGURE 2:**
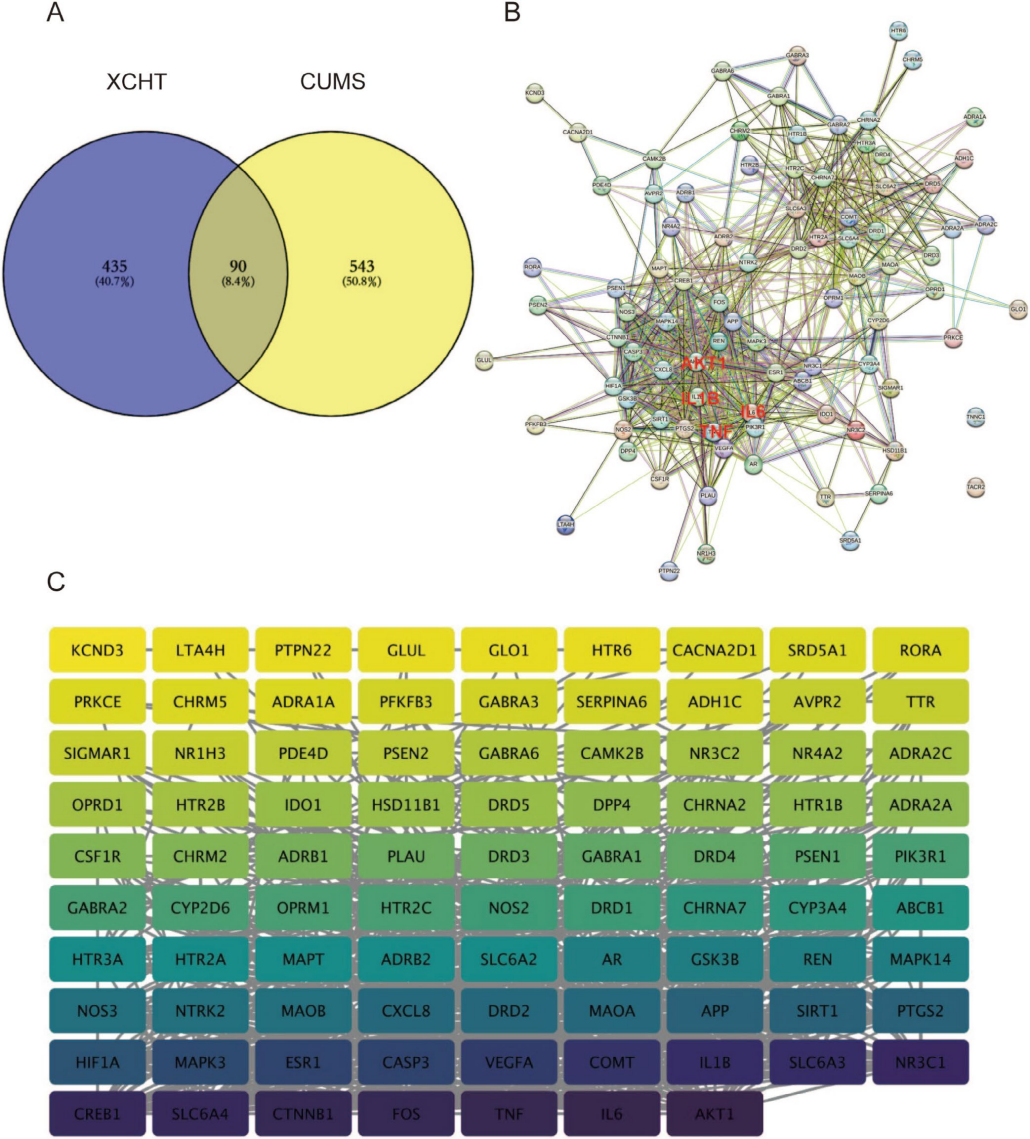
PPI Analysis of Targets of XCHT against Depression. (A) Venn diagram of intersectional genes between XCHT and depression. (B) Protein–protein interaction (PPI) network constructed by STRING; (C) PPI network constructed by Cytoscape. Degree values are visualized by sizes and colors of the node. Larger and darker nodes indicate higher degree values.

As shown in Figure 2A, 633 depression‐related eliminate duplicate targets were obtained from the TTD, OMIM, Genecards, drugbank, and DisGeNET databases, respectively. After intersecting with XHCT‐related targets, a total of 90 common targets were obtained. The network constructed by STRING contained 90 nodes and 710 edges (Figure 2B), and 2 distinct target clusters are evident in the figure; one is inflammation‐related, and the other is neurotransmitter receptor‐related. Then, the network constructed in Cytoscape contained 88 interconnected nodes (Figure 2C), with AKT1 (degree = 78), IL‐6 (degree = 76), TNF (degree = 74), CTNNB1 (degree = 72), and FOS (degree = 72) having the top 5 highest degrees.

### 3.4 GO and KEGG Analysis

**FIGURE 3:**
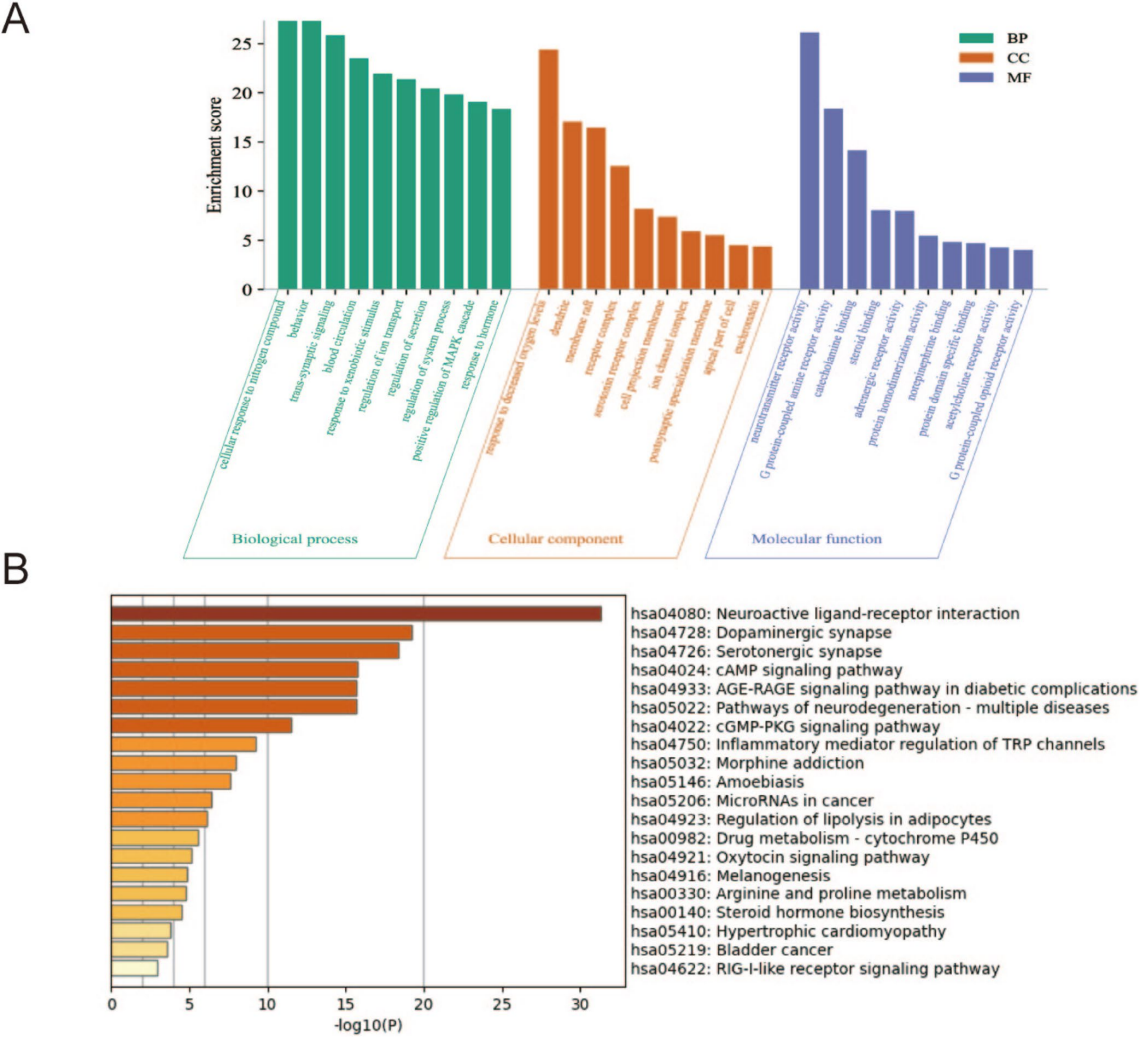
GO, KEGG analysis, and molecular docking. (A) GO enrichment analysis. Top 10 significantly enriched GO terms in biological process (BP), cellular component (CC), and molecular function (MF) are presented; (B) The top 20 pathways in KEGG enrichment analysis.

According to Metascape analytic results (Figure 3A), in the biological process, the screened targets were most abundant in the following biological processes, including “cellular response to nitrogen compound”, “trans‐synaptic signaling”, “behavior”, and “response to xenobiotic stimulus”. In the cellular component, the products of key target genes were mainly located in “synaptic membrane”, “dendrite”, “membrane raft”, “receptor complex”, and “serotonin receptor complex”. What's more, “neurotransmitter receptor activity” and “G protein‐coupled amine receptor activity” ranked the highest in the molecular function category.

The KEGG pathways were enriched by Metascape analysis (*p* < 0.05), and the top 20 enriched pathways are shown in Figure 3B. Among them, the inflammatory mediator regulation of TRP channels is closely related to the inflammatory response. Importantly, the total predicted pathways contain not only inflammatory response pathways in this study but also have several potential pathways related to the transmission and metabolism of neurotransmitters, such as the neuroactive ligand‐receptor interaction pathway, dopaminergic synapse pathway, and serotonergic synapse pathway. Combined with PPI network analysis, we considered that XCHT might alleviate depression by regulating the inflammatory response and metabolism of neurotransmitters.

### 3.5 Molecular Docking

**FIGURE 4:**
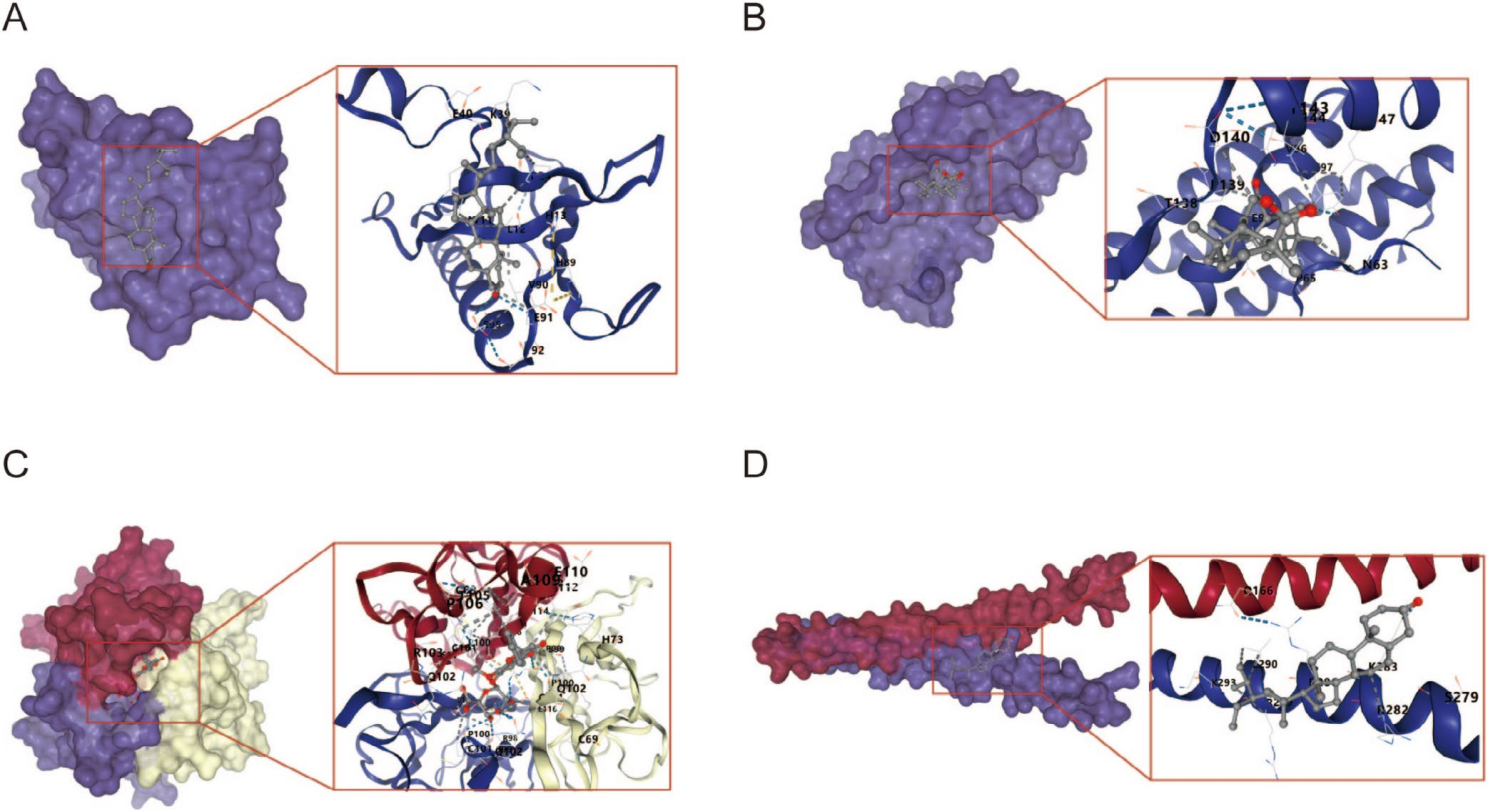
The binding modes between the top four targets and active compounds. (A) The molecular docking of AKT and beta‐sitosterol; (B) The molecular docking of IL‐6 and enoxolone; (C) The molecular docking of TNF and Wogonoside; (D) The molecular docking of FOS and beta‐sitosterol.

Five core components in the “drug‐component‐disease‐target” network (wogonin, wogonoside, enoxolone, stigmasterol) binding energy of 5 core compounds for molecular docking. As shown in Table 3 and Figure 4, all docking binding energies were less than −6.1 kcal·mol^−1^, indicating that the five core components and five key core targets had a good binding ability. Besides, there were 12 groups with binding energy less than −7.0 kcal·mol^−1^, indicating their strong binding activity. The above docking results fully show that the active ingredients in the XCHT can spontaneously bind to five core proteins to exert anti‐inflammatory activity.

**TABLE 3:** The binding energy of 5 core compounds with five key core targets.

Compound	Chemical formula	Binding energy with AKT1 kJ/mol^−1^	Binding energy with IL‐6 kJ/mol^−1^	Binding energy with TNF kJ/mol^−1^	Binding energy with FOS kJ/mol^−1^	Binding energy with CTNNB1 kJ/mol^−1^
Wogonin	C_16_H_12_O_5_	−6.2	−6.3	−8.4	−6.9	−6.1
Wogonoside	C_22_H_20_O_11_	−6.9	−6.4	−10.3	−7.5	−6.6
Enoxolone	C_30_H_46_O_4_	−6.9	−7.7	−8.8	−9.0	−6.7
Stigmasterol	C_29_H_48_O	−6.8	−7.0	−8.6	−7.4	−6.7
Beta‐Sitosterol	C_29_H_50_O	−7.1	−6.3	−8.2	−9.3	−6.7

### 3.6 Effects of XCHT on Depression‐Like Behavior and HPA Axis

**FIGURE 5:**
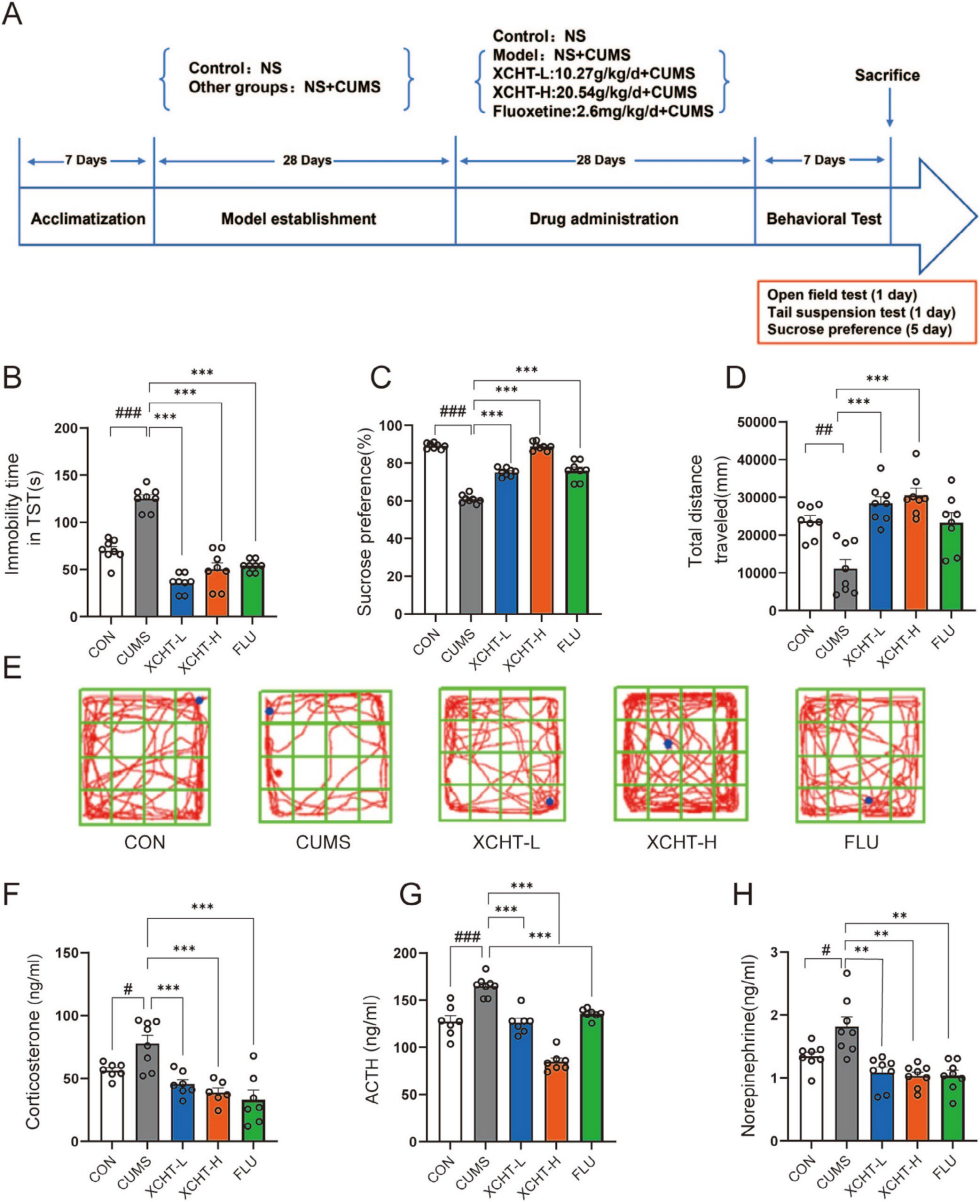
Effects of XCHT on mice depression‐like behavior, *n* = 8. (A) Schematic diagram of the animal experiments; (B) Immobility time in TST; (C) Sucrose preference in each group of mice; (D) The total distance of the mouse in the open field; (E) The representative trajectories of the mouse in the open field; (F) Level of corticosterone; (G) Level of ACTH; (H) Level of norepinephrine. The data were expressed as means ± SEM. ^#^ *p* < 0.05, ^##^ *p* < 0.01, ^###^ *p* < 0.001compared to CON group; **p < 0.01, ***p < 0.001 vs. CUMS group.

The schematic diagram of the animal experiments is shown in Figure 5A. In the TST (Figure 5B), compared to the control group, the CUMS group showed greater immobility time (*p* < 0.001). After 4‐week XHCT gavage, the mice in the XCHT‐L and XCHT‐H groups exhibited less immobility time (*p* < 0.001). The sucrose preference test is an indicator of the absence of responsive pleasure, which refers to a lack of interest in rewarding stimuli, a manifestation of an affective disorder, including depression. The XCHT can reverse the low sugar preference of mice that underwent CUMS (Figure 5C, *p* < 0.001), which demonstrated similar efficacy to the positive drug fluoxetine. Besides, in Figure 5D,F, XCHT can significantly reverse the depressive‐like behavior caused by CUMS and increase the total route distance explored by mice in the open field. Meanwhile, we determined levels of ACTH, CORT, and norepinephrine in the peripheral blood by ELISA to investigate whether XCHT treatment could alter the HPA axis and SNS hormones in depressed mice (Figure 5F–H). CUMS significantly increased two HPA axis hormones (ACTH and CORT) and norepinephrine in serum (both *p* < 0.05). XCHT could alter the effect (*p* < 0.05, *p* < 0.01, respectively).

### 3.7 XCHT Regulated Tryptophan Metabolism and Neurotransmitters in the Hippocampus in Mice

**FIGURE 6:**
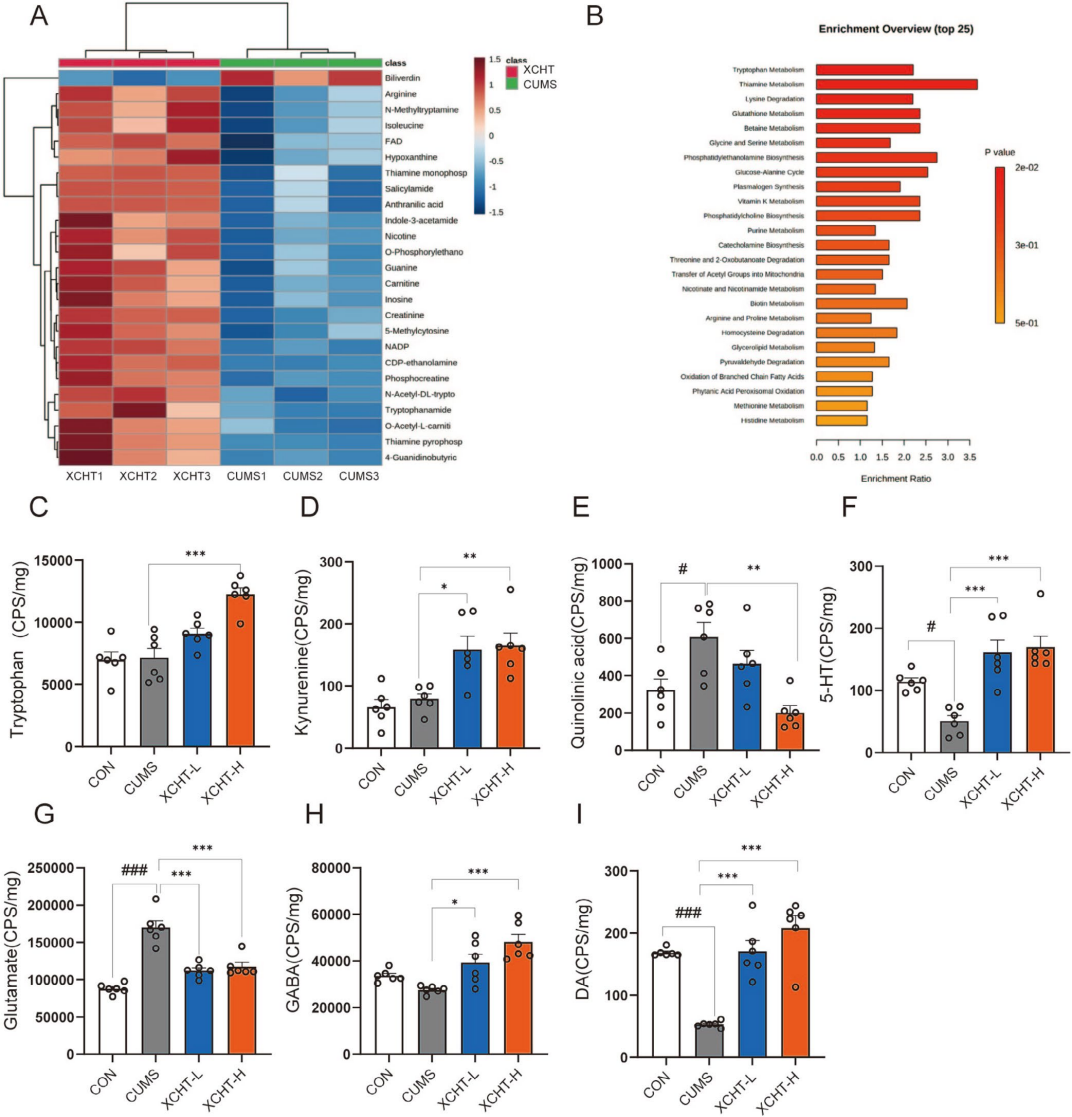
Effects of XCHT on the metabolism of hippocampal tissue, *n* = 6. (A) Heatmap of relationships between groups and hippocampus differential metabolites; (B) Enrichment analysis of KEGG pathway based on different metabolites in the hippocampus; XCHT significantly increased the levels of Trypotophan (C), Kynurenine (D), Quinolinic acid (E), 5‐HT (F), GABA (H), DA (I) in the hippocampal tryptophan metabolic pathway and neurotransmitters. XCHT decreased the levels of neurotransmitters: Glutamate (G). The data were expressed as means ± SEM. ^#^ *p* < 0.05, ^###^ *p* < 0.001, compared to CON group; *p < 0.05, **p < 0.01, ***p < 0.001 vs. CUMS group.

We demonstrated a clear separation of hippocampal tissue metabolites between the CUMS and XCHT groups, indicating that the samples in the CUMS group deviated from normal levels and exhibited metabolic disorders in Figure 6. The metabolites in the hippocampal tissue were identified based on MS data, KEGG, and HMDB 4.0. Potential biomarkers were screened according to the VIP value (> 1.0) and statistical tests (*p* < 0.05). The heatmap was used to illustrate the changes in metabolite levels between different groups in Figure 6A.

Importantly, the tryptophan‐related metabolites decreased, such as “Tryptophan amide”, “N‐Acetyl‐DL‐trypot”, and “N‐Methyltryptamine” metabolites. The KEGG pathway analysis is in Figure 6B, which shows that “Tryptophan Metabolism,” “Thiamine Metabolism,” “Lysine Degradation,” “Glutathione Metabolism,” and “Thiamine Metabolism” are the top 5 related pathways. Besides, compared with the CUMS model group, XCHT significantly increased the levels of tryptophan and kynurenine in hippocampal tissues and reduced the level of quinoline acid, a neurotoxic metabolite of tryptophan (Figure 6C–F). As well, the XCHT significantly increased the levels of GABA, and DA (both *p* < 0.01), and reduced the level of glutamate (*p* < 0.01) in Figure 6G–I.

### 3.8 Effects of XCHT on mRNA in the Hippocampus Based on RNA Sequencing

**FIGURE 7:**
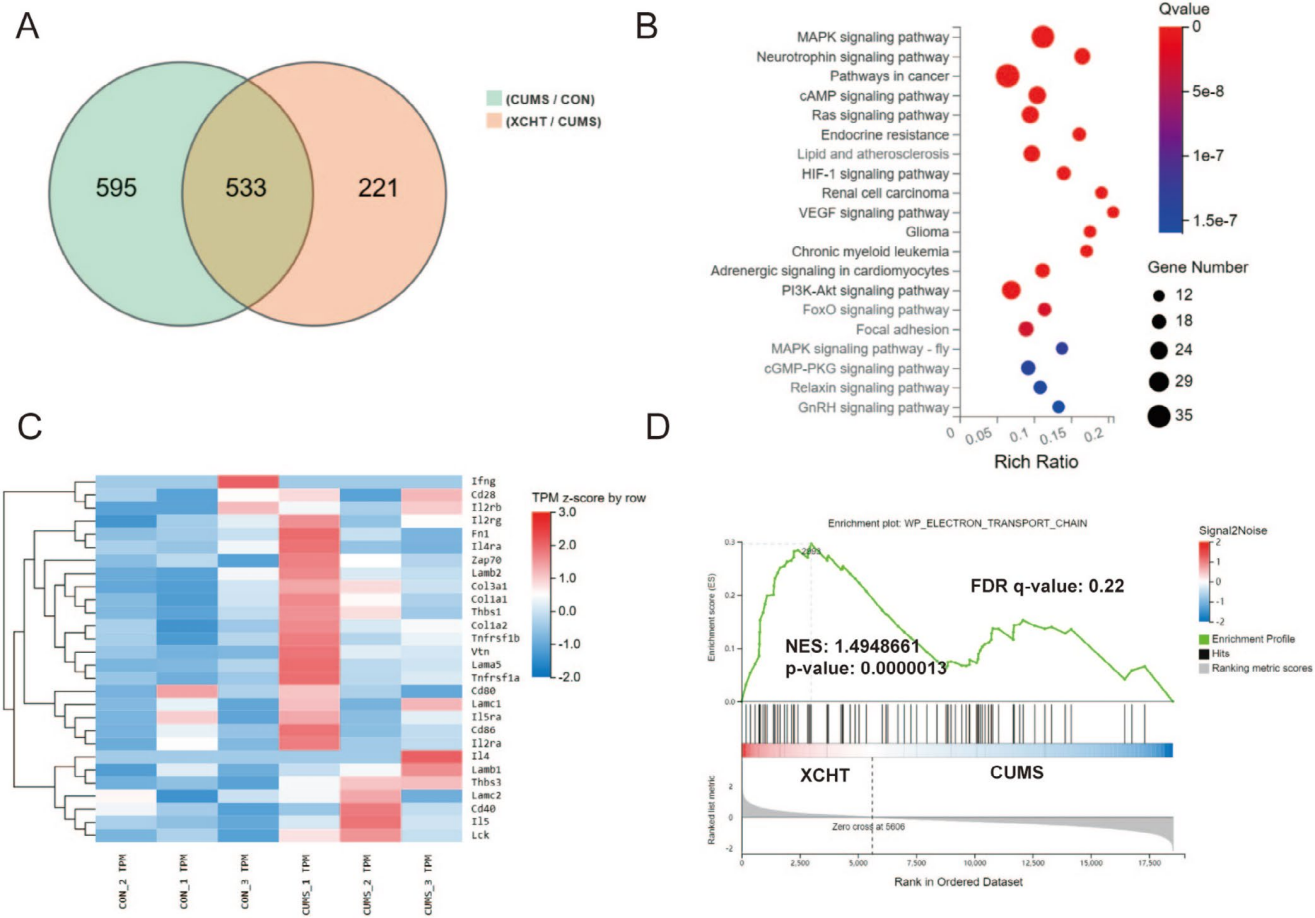
RNA sequencing (RNA‐seq) and bioinformatics analysis, *n* = 3. (A) Venn diagram of intersectional genes between the XCHT and CUMS groups; (B) The top 20 pathways in KEGG enrichment analysis between the XCHT and CUMS groups; (C) The top 28 differentially expressed genes analysis by heatmap between the CON and CUMS groups; (D) The typical GSEA analysis between the XCHT and CUMS groups.

We used RNA sequencing to investigate the effects of XCHT on the hippocampus in mice (Figure 7). Through RNA‐seq, we obtained 533 differential genes of the hippocampal in each group of mice, among which mRNAs such as Egr3, Dio2, Tsc22d3, Nfkbia, Pik3ca, Mapk11were upregulated in the XCHT group, while Pcdha4, Myt1, Slc9a2, and Atp11b were downregulated in the XCHT group (Figure 7A). The results of KEGG showed that the differential genes of the XCHT group were mainly enriched in the neurotrophin signaling pathway, mitogen‐activated protein kinase (MAPK), PI3K‐AKT signaling pathway, and other pathways (Figure 7B). Differential gene expression heatmaps (CON/CUMS) showed that the differential genes were mainly involved in neuroinflammation, microglia activity, and other processes (Figure 7C). Gene Set Enrichment Analysis (GSEA) was generated to show the electron transport chain involved in the effect of XCHT (Figure 7D).

### 3.9 Effects of XCHT on Hippocampal Microglial Activation and Gene Expression of Inflammatory Factors

**FIGURE 8:**
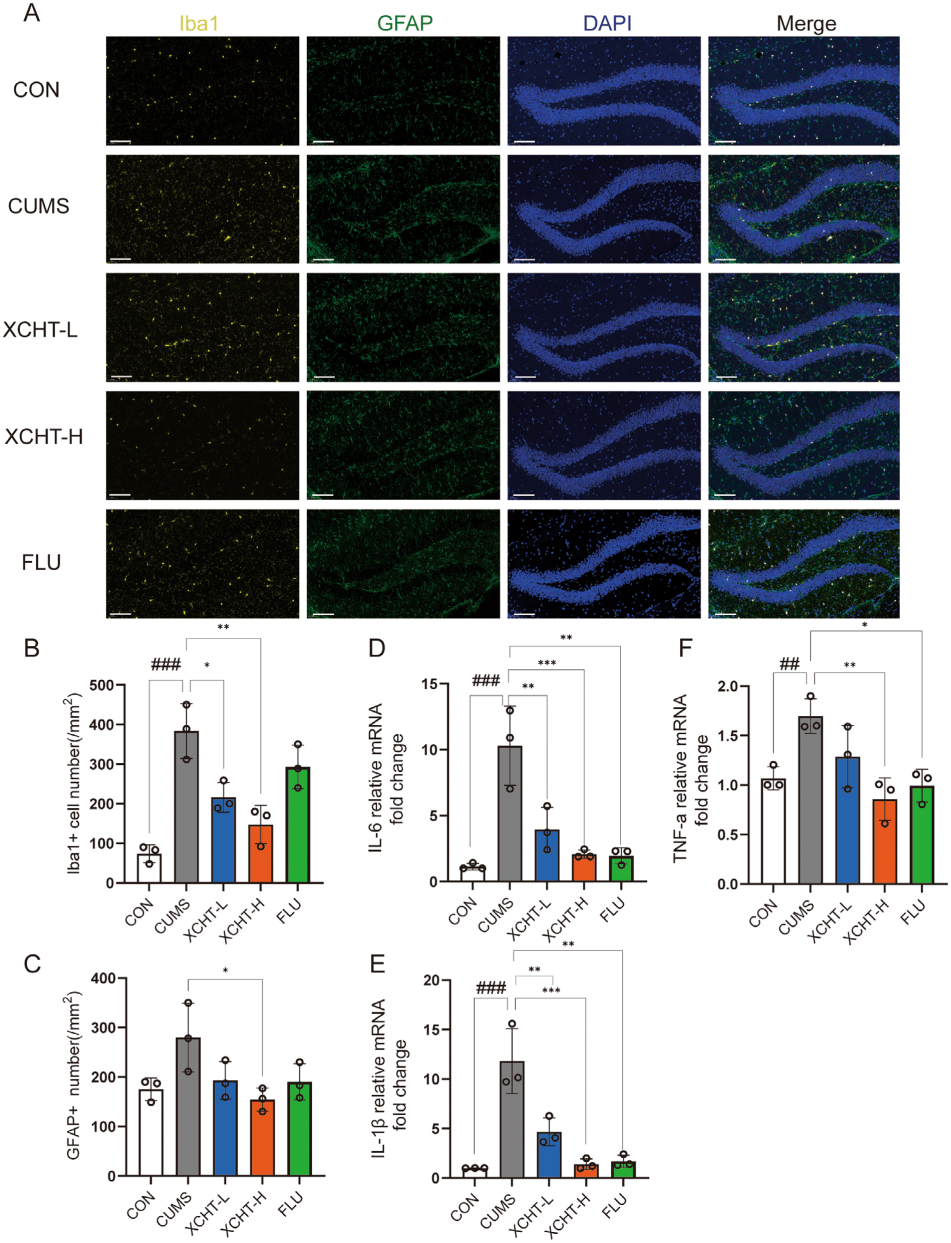
Effects of XCHT in regulating neuroinflammation, *n* = 3. (A) Activation of microglia in hippocampal tissues of DGs in different groups of mice. (gold: Iba1; green: GFAP; Blue: DAPI). ×20,100 μm. (B) Number of Iba1 labeled cells; (C) Number of GFAP labeled cells; (D) IL‐6, (E) IL‐1β, (F) TNF‐α mRNA levels in hippocampal tissues. ×20, 100 μm. ^##^ *p* < 0.01, ^###^ *p* < 0.001 compared to CON group; *p < 0.05, **p < 0.01, ***p < 0.001 vs. CUMS group.

Recently, neuroinflammation, characterized by microglial activation, has been suggested as a possible mechanism of stress‐induced depressive disorders. We observed the amount of Iba‐1‐labeled microglial cells, and GFAP‐labeled astrocytes was significantly larger in the hippocampus of stressed rats than in non‐stressed control mice (Figure 8A). XCHT significantly decreased the number of Iba‐1 and GFAP‐labeled microglial (Figure 8B,C, *p* < 0.001, *p* < 0.05, respectively). We then analyzed gene expression of inflammatory factors by RT‐PCR. The results showed that mRNA levels of IL‐6, IL‐1β, and TNF‐α (Figure 8D–F) decreased significantly in the hippocampus of the XCHT group compared with CUMS (*p* < 0.001, *p* < 0.001, *p* < 0.01, respectively).

### 3.10 XCHT Regulated Neuron Regeneration via BDNF/TrkB/CREB and PI3K/AKT Signaling Pathways

**FIGURE 9:**
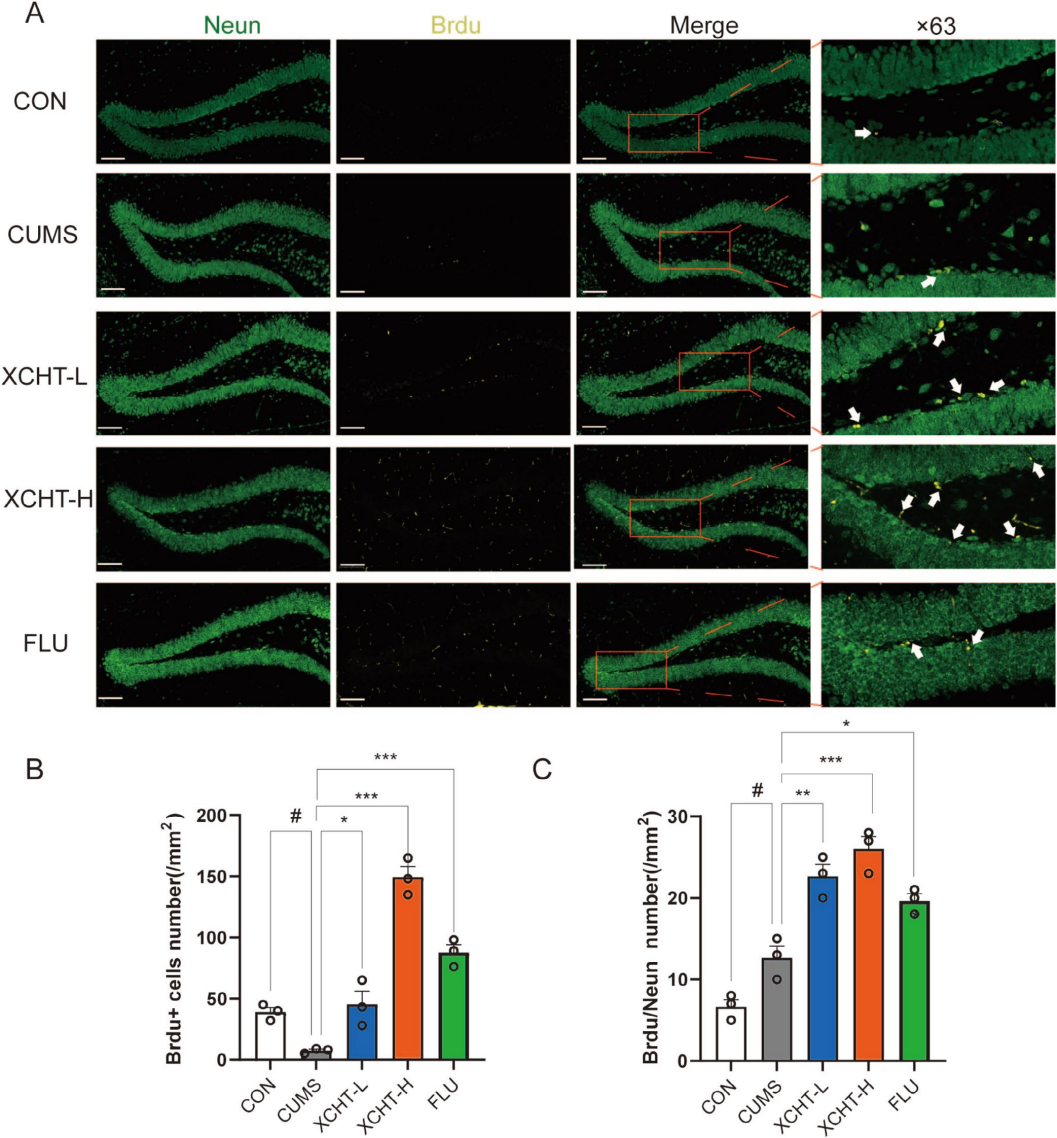
XCHT promoting nerve regeneration, *n* = 3. (A) Activation of BrdU in hippocampal tissues of DGs in different groups of mice. (gold: BrdU; green: Neun). (B) Number of BrdU/Neun labeled cells; (C) Number of BrdU labeled cells. ×20, ×63, 100 μm. ^#^ *p* < 0.05, compared to CON group; *p < 0.05, **p < 0.01, ***p < 0.001 vs. CUMS group.

Neurodegenerative disorders were reported to increase the occurrence of depression. We observed XCHT increased the DGs neurogenesis (Figure 9A), as demonstrated by a higher number of ^+^BrdU–NeuN^+^ newborn neurons (Figure 9B) as well as increased the number of BrdU single positive labeled cells (Figure 9C) in XCHT mice compared with stressed mice (*p* < 0.001, *p* < 0.001, respectively).

**FIGURE 10:**
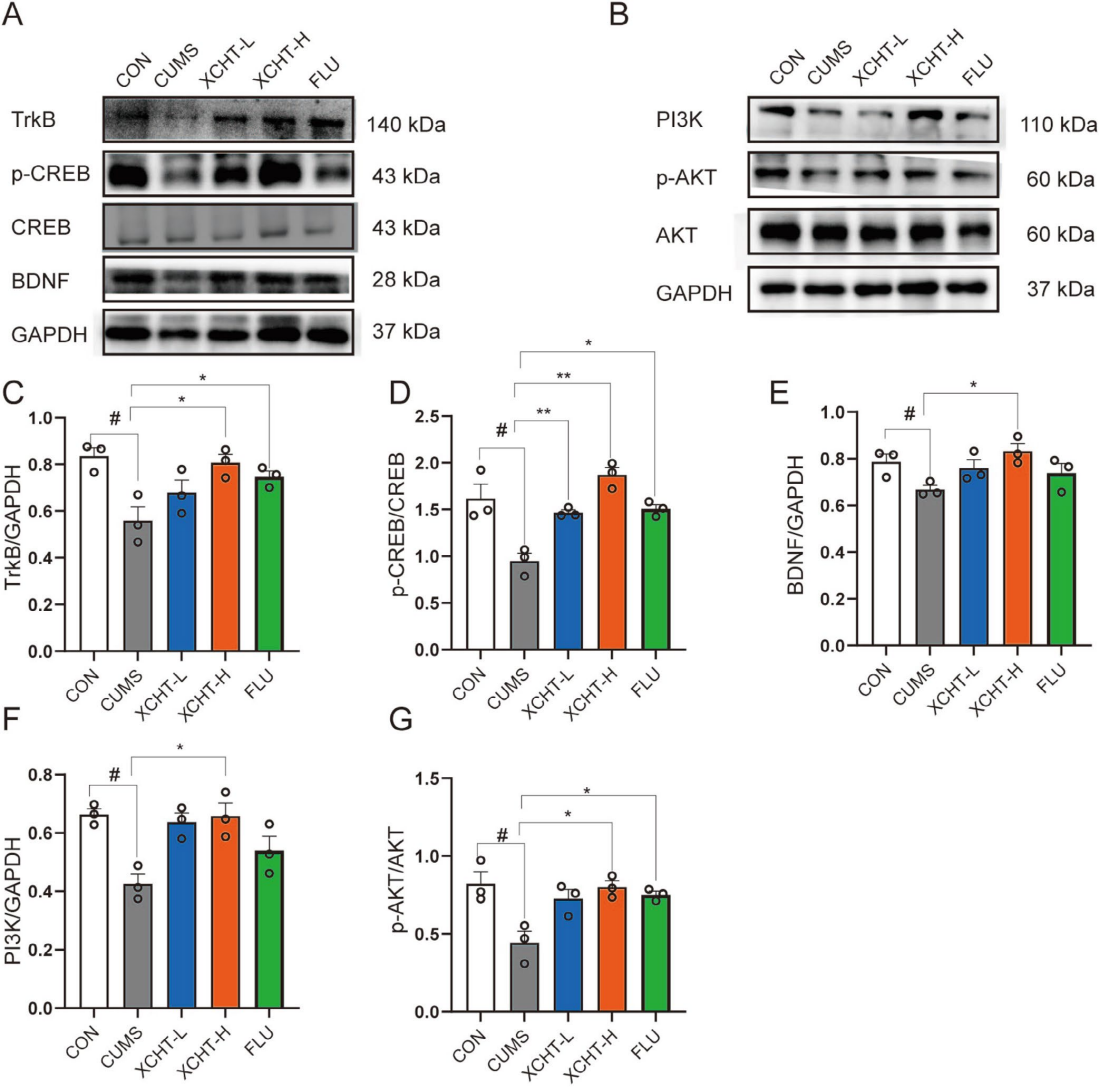
XCHT upregulated the mouse hippocampal BDNF/TrkB/CREB and PI3K/AKT signaling pathways, *n* = 3. (A) and (B) Representative band plot for each protein and statistical results of relative protein expression of TrkB (C), p‐CREB/CREB (D), BDNF (E), PI3K (F) and p‐AKT/AKT (G). The data were expressed as means ± SEM. ^#^ *p* < 0.05, compared to CON group; *p < 0.05, **p < 0.01, vs. CUMS group.

As shown in Figure 10A,B, A western blotting was used to validate the effects of XCHT on BDNF/TrkB/CREB and PI3K‐AKT signaling pathways in the hippocampus of mice. XCHT significantly increased the expression of BDNF, TrkB, p‐CREB, PI3K, and p‐AKT (Figure 10C–G, *p* < 0.01, *p* < 0.05, respectively).

## 4 Discussion

Chronic stress is a widespread trigger for depression, leading to a host of neurobiological changes that drive the development of this mental health condition [41, 42]. Despite advancements in this field, the underlying mechanisms of depression and its treatment remain largely enigmatic. While several antidepressant western drugs have been developed, they often lack efficacy and can cause numerous side effects. This has led to a growing interest in exploring alternative and complementary treatments, particularly traditional chinese medicine, as a means to address this pressing public health issue [43]. The current study aims to investigate the mechanisms of action of XCHT, a well‐known chinese traditional medicine, in the treatment of depression. Our study used combined network pharmacology, molecular docking, multi‐omics, and experimental validations to determine the effects of XCHT on depression‐related behavioral changes, potential active ingredients, and the underlying molecular mechanisms. The study will also determine the potential of XCHT as a new target for the development of antidepressants in the brain.

In our study, we used UPLC‐Q‐TOF‐MS to identify XCHT components that can enter the brain, and we used network pharmacology to identify the main mechanisms by which XCHT may act. KEGG pathway and GO analysis in network pharmacology showed XCHT could alleviate depression via mediating the inflammatory response, neuroactive ligand‐receptor interaction, and serotonergic synapse, which were also observed in non‐targeted metabolism results and RNA‐seq results. Through behavioral studies, we discovered that XCHT could improve depression in mice, increase the tryptophan metabolic pathway, and re‐regulate the HPA axis in vivo animal studies. We confirmed that XCHT could improve depression‐induced Iba‐1 expression and microglial activation in a subsequent immunofluorescence study. XCHT could reduce the formation of pro‐inflammatory cytokines in the hippocampus of depressed mice as well as the expression of BrdU+. Meanwhile, western blot also revealed the BDNF/TrkB/CREB and PI3K/AKT signaling pathways were increased by XCHT, resulting in neurogenesis.

Neuroinflammation mediated neuronal damage was the crucial pathological characteristic of depression. IL‐1β pathway upregulated by microglia may activate M1‐like microglial and A1‐like astrocytes, resulting in neuron damage and synaptic injury via activating NLRP3 [44]. M1‐like microglia marked with CD80 and CD86 highly express many neurotoxic genes, such as IL‐1β, IL‐6, TNF‐α, IFN‐γ and inducible NO (iNOS) [45], which were observed in our study. Tryptophan (Trp) metabolites produced by commensal flora were reported to regulate microglial activation and TGF and VEGF‐B production, modulating astrocyte transcriptional programs and CNS inflammation via a mechanism mediated by the aryl hydrocarbon receptor (AHR) [46]. Tryptophan metabolism is important in regulating inflammation in the brain. When there is inflammation in the brain, immune cells release cytokines, which can activate the enzyme indoleamine 2,3‐dioxygenase (IDO) in astrocytes. IDO metabolizes tryptophan into kynurenine and other metabolites, which have immune‐regulatory effects and can modulate the activity of immune cells [47]. However, excessive activation of IDO and the kynurenine pathway can also be detrimental to the brain, leading to neuroinflammation and neurotoxicity [48]. This is because kynurenine metabolites, particularly quinolinic acid, can induce oxidative stress and damage neurons [49]. In our study, XCHT was proven to alleviate depressive disorders by downregulation of IL‐1β, TNF‐α, and IL‐6 inflammasome, modulating the immune system by inhibiting Iba‐1 and GFAP overexpression, making it a promising candidate for the treatment of depression. Overexpression of pro‐inflammatory cytokines is the etiology of the imbalance of kynurenine and tryptophan metabolism. Another function of microglia and astrocytes is to control the levels of neurotransmitters in the brain, including serotonin, which is synthesized from the amino acid tryptophan [50]. In our study, the tryptophan metabolic pathway, indole‐3‐acetamide, and the neurotransmitters (5‐HT, GABA, DA) were upregulated by XCHT, which may lead to anti‐neuroinflammation, alleviating depression disorders. Meanwhile, fast chemical communication in the nervous system is mediated by neurotransmitter‐gated ion channels, which were demonstrated in the Gene set enrichment analysis (GESA); the electric transport chain has been upregulated by XCHT.

Chinese medicine has a long history of treating “Yu” syndrome, often acting through herbal medicines such as tonifying the liver and Qi, strengthening the spleen, and calming the mind [51, 52]. In the previous study, the Chai Hu formula, like Chai‐Hu‐Shu‐Gan‐San, can treat depression by reversing stress‐induced disruption of ERK1/2 and ERK5 activity, a novel member of the MAPKs family, and is also known as the big MAP kinase, belonging to neuroactive ligand‐receptor interaction [53, 54, 55]. Meanwhile, the BDNF‐ERK‐CREB pathway was reported to be upregulated by Chai‐Hu‐Shu‐Gan‐San, leading to cognitive improvement and anti‐depression [56]. BDNF stands for Brain‐Derived Neurotrophic Factor, which is a protein that is critical for the growth, development, and maintenance of neurons in the brain and nervous system [57]. BDNF plays a crucial role in many important functions, such as learning and memory, as well as regulating mood and emotions [58]. Similar to Chai‐Hu‐Shu‐Gan‐San, XCHT was reported to attenuate depressive/anxiety‐like behaviors of social isolation‐reared mice by regulating the monoaminergic system, neurogenesis, and BDNF expression. Otherwise, the expression of BDNF has crucial relationships with neuroinflammation [59, 60]. In our study, the computational network pharmacology strategy revealed that key functional components were ginsenosides, baicalins, saikosaponins, and glycyrrhizic acids. They were all reported to show anti‐depression function by downstream inflammatory pathways, such as NF‐kB, MAPKs, PI3Ks, and upregulation of BDNF expression with neuronal repairment [61, 62, 63, 64, 65]. Protein kinases such as PI3K/Akt and ERK/MAPK are activated by BDNF–TrkB signaling [66]. In our study, XCHT could promote neurogenesis via BDNF/TrkB/CREB and PI3K/AKT signaling pathways. While, our study exist several limitations; gender differences in depression need to be observed further. Female cases with depression were much higher than male cases, also a number of studies reported greater efficacy of selective serotonin reuptake inhibitors in women and better therapeutic response of the tricyclic antidepressant imipramine in men [67, 68, 69]. The mechanisms of gender differences in depression were ambiguous. Although XCHT demonstrated some antidepressant effects in perimenopausal depressed mice [12], the exact mechanism remains unclear. The promising mechanism may be related to XCHT reducing neuroinflammation by alleviating the brain inflammatory environment. Overall, targeting BDNF/TrkB/CREB and PI3K/AKT signaling pathways or their upstream pathways may offer potential therapeutic strategies for the treatment of neuroinflammatory and neurodegenerative diseases.

## 5 Conclusion

The results of the current study suggest that XCHT may have a role in the treatment of depression by modulating the immune system and reducing neuroinflammation in the brain. Our study also highlights the potential of XCHT as a new target for the development of antidepressants in the brain. These findings contribute to the understanding of the mechanisms underlying depression and its treatment, and provide a foundation for further research into the therapeutic potential of TCM in the treatment of depression.

## Author Contributions

Y.F., W.K.W., and Y.R.Z. conceptualized the manuscript. Y.F. and W.K.W. wrote the manuscript. Y.R.Z. and Y.Y.Z. edited the critical revisions. Y.F. and W.K.W. performed the analyses. Y.F. and Y.R.Z. carried out the painting of graphics. W.K.W., Y.Y.F., and Z.Z.Z. participated in the design of experiments and animal rearing and provided guidance on article writing. Y.W. provided supervision of the entire manuscript. All authors approved the final version of the manuscript for submission.

## Conflicts of Interest

The authors declare no conflicts of interest.

## Declaration of Generative AI in Scientific Writing

The authors declare not to use AI tools to analyze and draw insights from data as part of the research process.

## Supporting information


Appendix S1.

## Data Availability

The data that support the findings of this study are available from the corresponding author upon reasonable request.
