# Supplementary material for: Xiao‐Chai‐Hu‐Tang Ameliorates Depressive Symptoms via Modulating Neuro‐Endocrine Network in Chronic Unpredictable Mild Stress‐Induced Mice
Source: CNS Neurosci Ther. 2025 Feb 21;31(2):e70290. doi: 10.1111/cns.70290 (PMC11843474; doi:10.1111/cns.70290)
Supplement: Supplementary file 1 — Appendix S1. [file CNS-31-e70290-s001.docx]

Table. S1 Mass parameters (Sciex Triple TOF 4600 LC-MS)

| MS parameter | Parameter value | MS/MS parameter | Parameter value |
| --- | --- | --- | --- |
| TOF mass range | 50～1700 | MS/MS mass range | 50～1250 |
| Ion Source Gas 1（psi） | 50 | Declustering Potential（V） | 100 |
| Ion Source Gas 2（psi） | 50 | Collision Energy（eV） | ±40 |
| Curtain Gas（psi） | 35 | Collision Energy Spread（eV） | 20 |
| Ion Spray Voltage Floating (V) | -4500/5000 | Ion Release Delay（ms） | 30 |
| Ion Source Temperature (°C) | 500 | Ion Release Width（ms） | 15 |
| Declustering Potential（V） | 100 |  |  |
| Collision Energy（eV） | 10 |  |  |


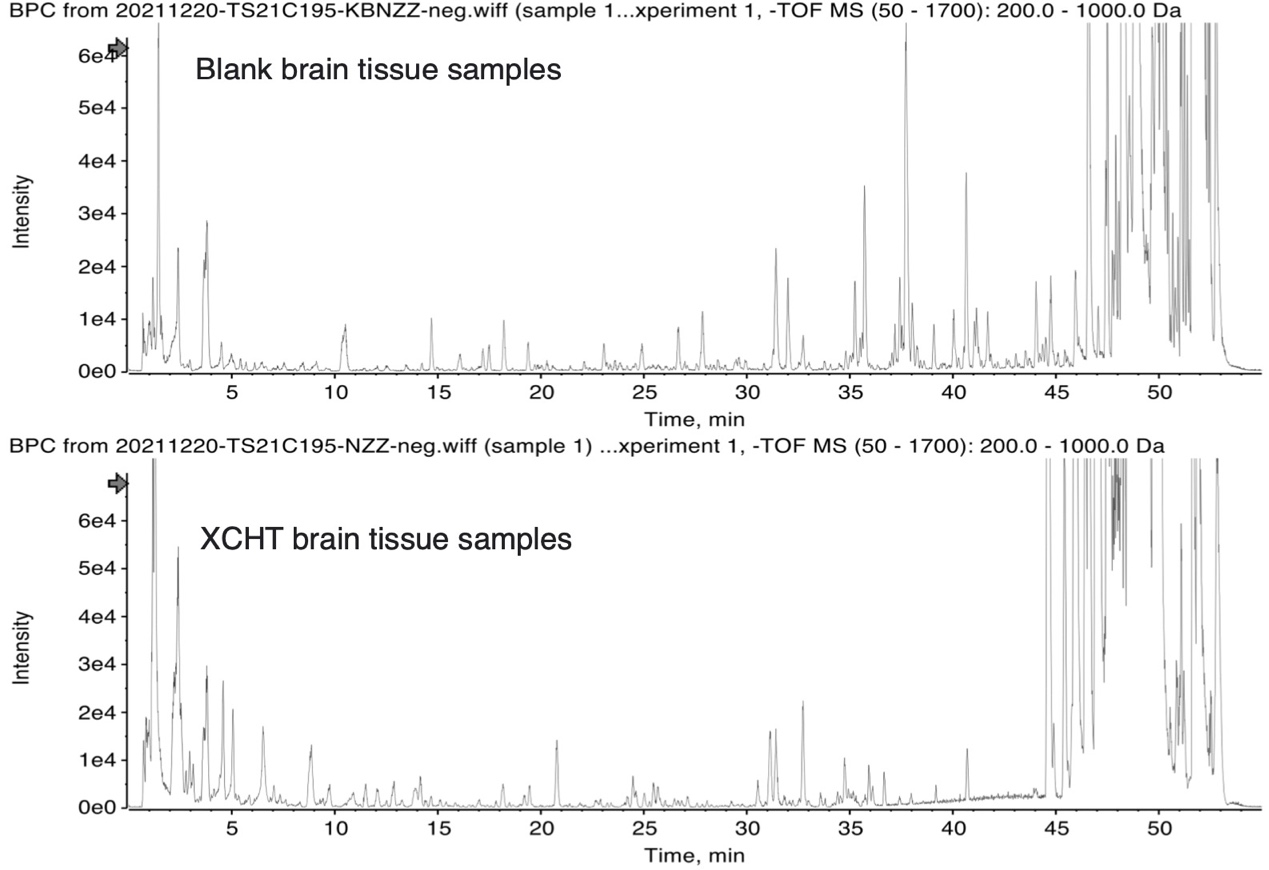


Figure. 1 Ion current plot (BPC)-negative ion pattern of UPLC-HRMS base peak in brain tissue samples


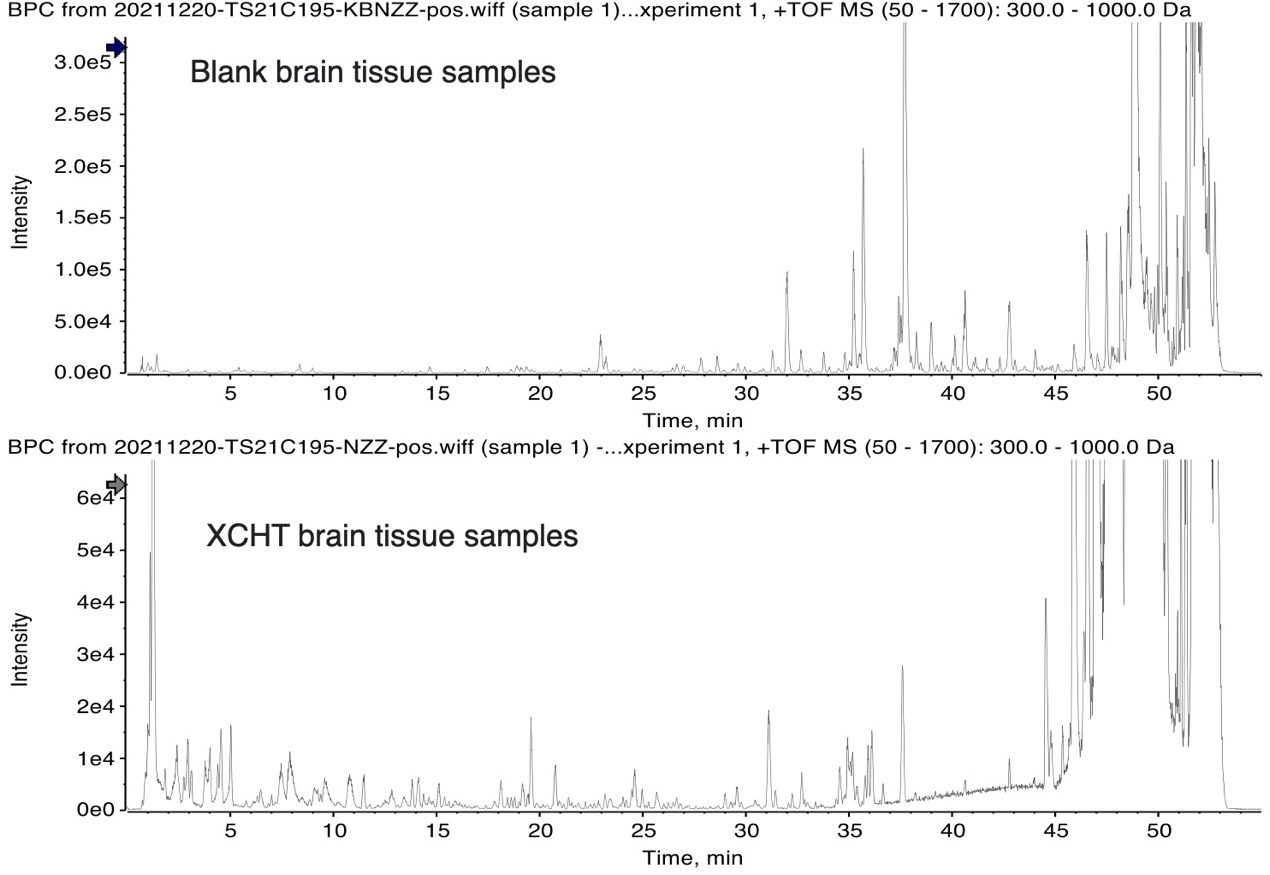


Figure. 2 Ion current plot (BPC)-positive ion pattern of UPLC-HRMS base peak in brain tissue samples

Table S2: Identification of prototype components of compound origin in brain tissue samples

| NO. | Time  (min) | Adduct Ion | ppm | Molecular Formula | Molecular Weight | Name | MS/MS data |
| --- | --- | --- | --- | --- | --- | --- | --- |
| 1 | 13.94 | [M-H]^-^ | -1.4 | C_26_H_30_O_13_ | 550.17 | Liquiritin apioside | 549.1598; 255.0660;135.0087;119.0502 |
| 2 | 14.13 | [M-H]^-^ | -1.1 | C_26_H_28_O_13_ | 548.15 | Chrysin 6-C-glucoside-8-C-arabinoside | 547.1455;487.1222;457.1116;427.1031;367.0816;337.0713 |
| 3 | 15.41 | [M-H]^-^ | -1.1 | C_26_H_28_O_13_ | 548.15 | Chrysin 6-C-D-arabinoside-8-C-glucoside | 547.1439;457.1112;427.1019;367.0785;337.0699 |
| 4 | 16.23 | [M-H]^-^ | -1.7 | C_26_H_28_O_13_ | 548.15 | Chrysin 6-C-hexoside-8-C-pentoside | 547.1457;457.1168;367.0797;337.0681;309.0743 |
| 5 | 17.37 | [M-H]^-^ | -0.2 | C_22_H_20_O_12_ | 476.10 | 5,7,2′-trihydroxy-6-methoxyflavone-7-O-glucuronide | 475.0869;299.0551; |
| 6 | 20.77 | [M-H]^-^ | -3.7 | C_21_H_18_O_11_ | 446.08 | Baicalin | 269.0437;223.0383 |
| 7 | 22.59 | [M-H]^-^ | -1.1 | C_22_H_20_O_11_ | 460.10 | Wogonoside isomer | 459.0925;283.0602;268.0379 |
| 8 | 22.76 | [M-H]^-^ | -1.1 | C_23_H_22_O_12_ | 490.11 | 5,7-Dihydroxy-8,2'-dimethoxyflavone7-O-β -D-glucuronide isomer | 489.1057;313.0721;298.0478;283.0218 |
| 9 | 22.88 | [M-H]^-^ | 0.4 | C_21_H_18_O_11_ | 446.08 | Wogonin5-O-β-D -glucoside | 269.0446;197.0586 |
| 10 | 23.06 | [M+FA-H]^-^ | -0.5 | C_42_H_72_O_14_ | 800.49 | Ginsenoside Rg1 | 845.4905;799.4838;637.4302;619.4109 |
| 11 | 23.53 | [M-H]^-^ | -2.7 | C_22_H_20_O_12_ | 476.1 | 5,7,8-trihydroxy-6-methoxyflavone-7-O-glucuronide | 475.0835;299.0536;284.0293 |
| 12 | 24.07 | [M-H]^-^ | -3.2 | C_21_H_18_O_11_ | 446.08 | Norwogonin 8-O-β-D-glucuronide | 445.0778;269.0458;197.0608 |
| 13 | 24.19 | [M-H]^-^ | -2.4 | C_21_H_18_O_10_ | 430.09 | Chrysin-7-O-β-D-glucoronide | 253.0488 |
| 14 | 24.48 | [M-H]^-^ | 2.9 | C_22_H_20_O_12_ | 476.1 | Diosmetin 7-O-β-D-glucuronide | 475.0880;299.0557;284.0320;268.0364 |
| 15 | 24.56 | [M-H]^-^ | -2.8 | C_22_H_20_O_11_ | 460.1 | Oroxylin A 7-O-glucuronide | 283.0601;268.0354 |
| 16 | 25.02 | [M-H]^-^ | -3.7 | C_21_H_18_O_11_ | 446.08 | Baicalein 6-O-β-D-glucuronide | 445.0758;269.0447;241.0496 |
| 17 | 25.69 | [M-H]^-^ | 4.2 | C_22_H_20_O_11_ | 460.1 | Wogonoside | 283.0603;268.0378 |
| 18 | 26.52 | [M-H]^-^ | 4.8 | C_23_H_22_O_12_ | 490.11 | 5,7-Dihydroxy-8,2'-dimethoxyflavone7-O-β -D-glucuronide isomer | 313.0632;298.0484;283.0223 |
| 19 | 27.99 | [M-H]^-^ | -1.3 | C_23_H_22_O_12_ | 490.11 | 5,7-Dihydroxy-8,2'-dimethoxyflavone7-O-β -D-glucuronide isomer | 313.0710;298.0473;283.0235 |
| 20 | 30.02 | [M+FA-H]^-^ | -2.3 | C_42_H_72_O_14_ | 800.49 | Ginsenoside Rf | 799.4862;637.4328;475.3825; |
| 21 | 32.21 | [M-H]^-^ | -2.4 | C_42_H_62_O_17_ | 838.4 | Licorice saponin G2 | 837.3928;351.0552 |
| 22 | 33.82 | [M+FA-H]^-^ | 2.6 | C_54_H_92_O_23_ | 1108.6 | Ginsenoside Rb1 | 1153.5984;1107.5926;945.5452;783.4937;621.4324 |
| 23 | 34.53 | [M+FA-H]^-^ | 1 | C_53_H_90_O_22_ | 1078.59 | Ginsenoside Rb2 | 1123.5926;1077.5874;945.5383;783.4898;621.4365 |
| 24 | 34.59 | [M-H]^-^ | 0.3 | C_48_H_76_O_19_ | 956.5 | Ginsenoside Ro | 955.4919;731.4456 |
| 25 | 34.67 | [M-H]^-^ | 0.4 | C_42_H_62_O_17_ | 838.4 | Yonuganoside K2 | 837.3947; 351.0570 |
| 26 | 35.24 | [M+FA-H]^-^ | 1.7 | C_53_H_90_O_22_ | 1078.59 | Ginsenoside Rb3 | 1123.5929;1077.5867 |
| 27 | 35.54 | [M-H]^-^ | 0.6 | C_42_H_62_O_17_ | 838.4 | Uralsaponin U | 837.3883; 351.0571 |
| 28 | 36.13 | [M-H]^-^ | -2.1 | C_16_H_12_O_5_ | 284.07 | Wogonin | 283.0645;268.0345;163.0021 |
| 29 | 36.68 | [M-H]^-^ | 0.1 | C_42_H_62_O_16_ | 822.4 | Glycyrrhizin | 821.3985;351.0563 |
| 30 | 37.43 | [M-H]^-^ | -0.3 | C_16_H_12_O_5_ | 284.07 | Wogonin isomer | 283.0623;268.0379;184.0539;163.0067 |
| 31 | 37.66 | [M+FA-H]^-^ | -1.4 | C_42_H_68_O_13_ | 780.47 | Saikosaponin A | 825.4613;779.4611;617.4039 |
| 32 | 38.16 | [M-H]^-^ | -0.9 | C_42_H_62_O_16_ | 822.4 | Uralsaponin B | 821.3958;351.0532;193.0327 |
| 33 | 38.18 | [M+FA-H]^-^ | -1.4 | C_42_H_68_O_13_ | 780.47 | Saikosaponin B2 | 825.4657;779.4586;617.4066 |
| 34 | 39.45 | [M+FA-H]^-^ | 1 | C_44_H_70_O_14_ | 822.48 | 2"-O-acetylsaikosaponin A | 867.4760;821.4613;779.4579;617.4101 |
| 35 | 39.85 | [M+FA-H]^-^ | -2.2 | C_42_H_68_O_13_ | 780.47 | Saikosaponin D | 825.4615;779.4560;617.4031 |
| 36 | 41.71 | [M+FA-H]^-^ | -2.7 | C_44_H_70_O_14_ | 822.48 | 4"-O-acetylsaikosaponin D | 821.4735;779.4582;617.4051 |
| 37 | 49.69 | [M-H]^-^ | -0.3 | C_30_H_46_O_4_ | 470.34 | Glycyrrhetin or isomer | 469.3323;425.3408;409.3103;355.2625 |
| 38 | 49.76 | [M-H]^-^ | 0.1 | C_30_H_46_O_4_ | 470.34 | Glycyrrhetin or isomer | 469.3291;425.3385;409.3065;355.2585 |


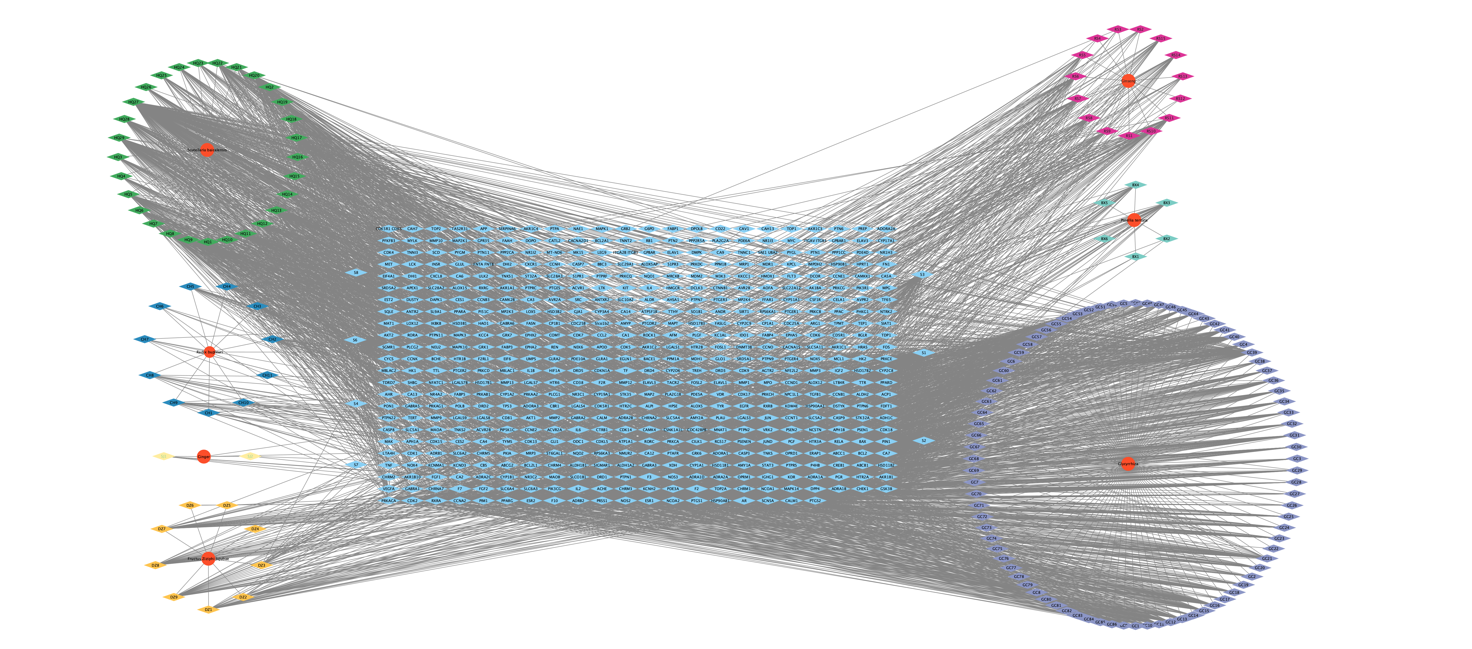


Figure. 3 "Herbs-Compounds-Targets" network of XCHT
